# Signatures of Chemical Dopants in Simulated Resonance Raman Spectroscopy of Carbon Nanotubes

**DOI:** 10.1021/acs.jpclett.2c03591

**Published:** 2023-01-30

**Authors:** Braden M. Weight, Ming Zheng, Sergei Tretiak

**Affiliations:** † Department of Physics and Astronomy, University of Rochester, Rochester, New York 14627, United States; ‡ Center for Integrated Nanotechnologies, Center for Nonlinear Studies, and Theoretical Division, Los Alamos National Laboratory, Los Alamos, New Mexico 87545, United States; § Materials Science and Engineering Division, National Institute of Standards and Technology, Gaithersburg, Maryland 20899, United States

## Abstract

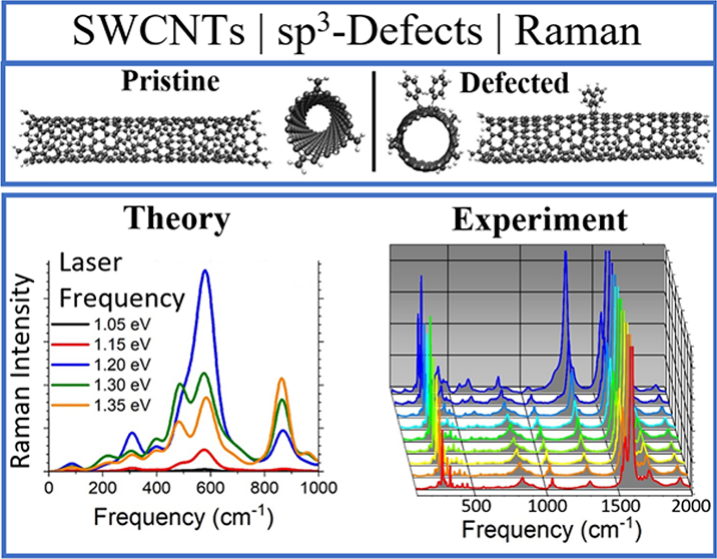

Single-walled carbon nanotubes (SWCNTs) with organic sp^2^ or sp^3^ hybridization defects allow the robust tunability of many optoelectronic properties in these topologically interesting quasi-one-dimensional materials. Recent resonant Raman experiments have illuminated new features in the intermediate-frequency region upon functionalization that change with the degree of functionalization as well as with interactions between defect sites. In this Letter, we report *ab initio* simulated near-resonant Raman spectroscopy results for pristine and chemically functionalized SWCNT models and find new features concomitant with experimental observations. We are able to assign the character of these features by varying the frequency of the external Raman laser frequency near the defect-induced E_11_* optical transition using a perturbative treatment of the electronic structure of the system. The obtained insights establish relationships between the nanotube atomistic structure and Raman spectra facilitating further exploration of SWCNTs with tunable optical properties tuned by chemical functionalization.

Chemical control of surface modification in single-walled carbon nanotubes (SWCNTs) has recently taken a leap forward after the development of new synthetic routes for functionalization of SWCNTs with single-stranded DNA (ssDNA):^1−3^ wrapping, photochemistry utilizing singlet and triplet state pathways,^4,5^ and well-established chemistries using diazonium salts, hypochlorite, and ozone functionalizations.^6−9^ In all of these cases, chemical functionalization^10−23^ creates a low-lying defect-associated E_11_* exciton state below the fundamental E_11_ exciton band of the SWCNT that exhibits improved optical properties and is energetically tunable.^7,14,24^ For example, in the case of ssDNA wrapping, it is hypothesized that the guanine nucleotide base uniquely reacts with the SWCNT surface to form a sp^3^ or sp^2^ hybridiztion defect with complete control over the spacing between the adjacent defects and hence their interdefect interactions and exciton delocalization properties. When utilizing photochemical routes, the photoexcited triplet pathway, in the absence of triplet-quenching oxygen species, has been shown to produce experimentally new atomistic configurations of defects expanding the diversity of bonding configurations on the SWCNT lattice. SWCNT hybridization defects, in general, are known to break the symmetry of the system and introduce novel, red-shifted, and localized excitons.^11,15,22,24−28^ Similar functionalized systems using a variety of chemical adducts (e.g., aryl, alkyl, etc.) have been well-studied both theoretically and experimentally. For example, the inductive effects of the chemical adduct^7,27,29−32^ and long- and short-range defect–defect interactions^33−35^ on the energetically low-lying defect-associated E_11_* exciton were shown to improve energetic tunability and the capability for single-photon emission.^36−41^ These studies have led to an improved understanding of these SWCNT systems and facilitated new experiments to deepen our understanding of these interesting topological materials.

Given a wide variety of possible defect configurations, identification of spectroscopic footprints of specific chemical groups bound to the tube surfaces, however, remains a challenge. For example, resonant Raman spectroscopy for pristine and functionalized SWCNTs has been reported^42−54^ but not well-decomposed and interpreted in the context of functionalization beyond the famous and prominent active modes in graphene and SWCNT systems, namely, the defect-associated mode (D), the graphene-like mode (G), and the radial breathing mode (RBM). These spectroscopic experiments are key to examining and explaining a great many things related to excited nonradiative dynamics, even for defect-free systems, relevant to transient^55−57^ and other nonlinear spectroscopies.^58^ Upon the introduction of local defects, these experiments probe processes such as exciton trapping/detrapping events at these trapping sites^33^ or exciton–polariton dynamics involving defect states.^16^

Our current work was primarily inspired by two experimental studies^2,29^ on defected SWCNTs. In both reports, E_22_-resonant Raman spectra of sets of defected SWCNTs were recorded upon application of ssDNA^2^ and aryl^29^ functionalizations. In both cases, and as expected for any SWCNT sample, the intensity of the primary D mode was increased compared to that of the G mode after surface functionalization. However, many interesting features in the intermediate regions of the Raman spectra between 300 and 1300 cm^–1^ were observed^2^ that may imply important features of these systems yet to be analyzed at the atomistic level. For example, these lower-energy modes may contribute to nonradiative relaxation processes involving transitions between excited states near the defect states that potentially can affect the physical properties of the material.

We point out that other groups have also reported resonance effects in SWCNT Raman spectroscopy in, for example, these intermediate-frequency regions^59^ of pristine SWCNTs as well as resonance effects on the D mode.^60^ The discussions were focused on multiparticle processes in mixed-chirality pristine samples^59^ and the nonlinear dependence of the intensity of the D mode based on negative or positive detuning of the excitation frequency from the main excitonic transition.^60^ Each report was supplemented with simulations but lacked explicit excitonic effects necessary in the description of excited electronic states in SWCNT systems. However, our results allow good comparison with each study for pristine intermediate modes and for the D mode resonance effects but yield a simpler interpretation for the formation of these additional features. These topics will be specifically discussed below.

Figure 1 showcases the two examples of resonant Raman spectra that form the inspiration and basis for this report. Figure 1a presents E_22_-resonant Raman spectra using data from the Supporting Information of ref (2) indicating similar features upon functionalization with ssDNA-based functional guanine species on the (9,1) SWCNT chirality. Figure 1b shows experimental E_22_-resonant Raman spectra using data from ref (29) varying the diazonium-based defect concentration (axis into the page) on the (6,5) SWCNT chirality. In the region between the radial breathing mode (RBM, ∼300 cm^–1^) and the D mode (∼1300 cm^–1^), termed the intermediate frequency mode (IFM)^61−67^ region, there are many peaks that undergo a change in intensity with an increase in defect concentration, most notably near 400, 600, and 1050 cm^–1^. In both experiments, the IFM region was shown to be strongly influenced by the presence of the surface defects. Additionally, the spacing between adjacent guanine base units in the ssDNA strand^2^ and the concentration of diazonium reagent^29^ directly influence the intensity of the IFM peaks, showing that the strengthened defect–defect interactions increase the intensity of these new modes with respect to the G mode.

**Figure 1:**
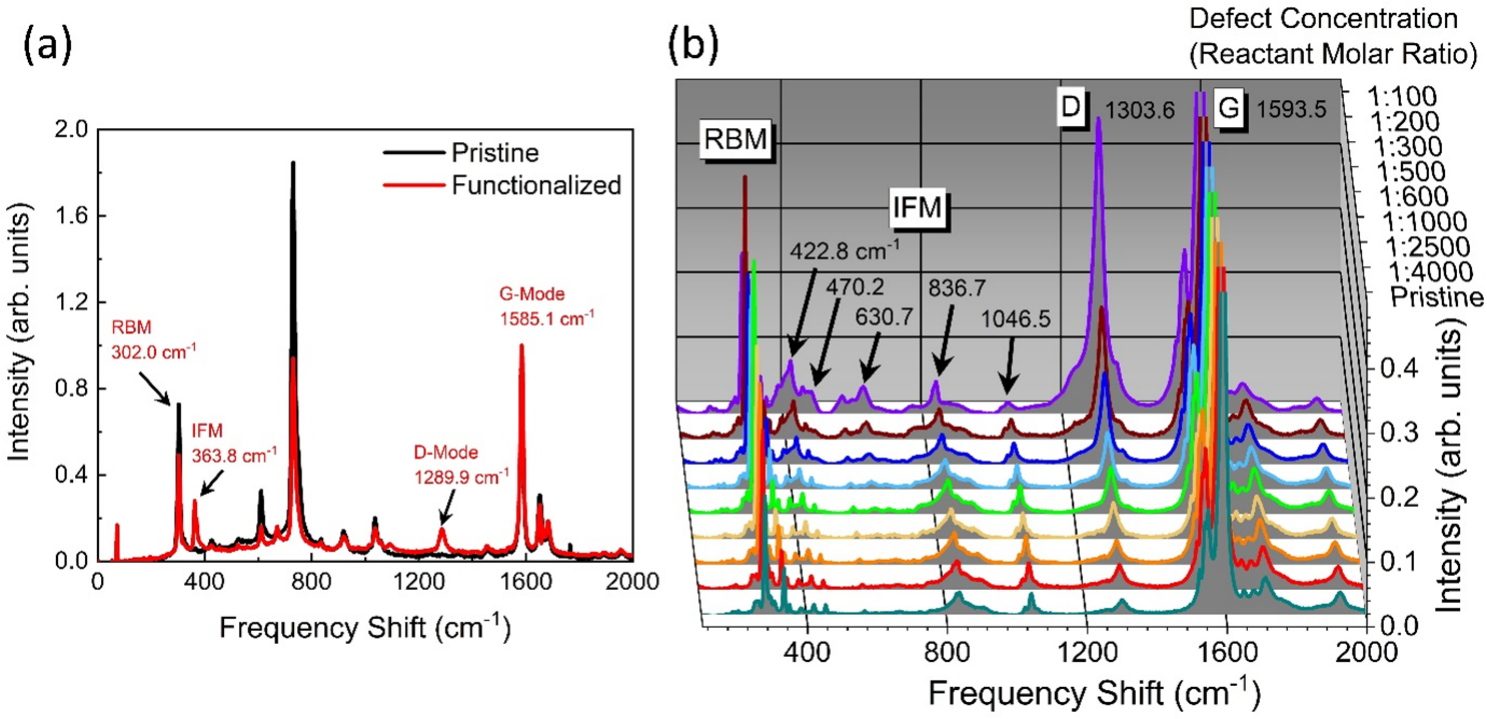
(a) Pristine (black) and functionalized (red) (9,1) SWCNT resonant Raman spectra measured in experiments. Here, the resonance frequency was introduced at 1.85 eV near the E_22_ band. (b) Resonant Raman spectra for varying amounts of surface functionalization on a (6,5) SWCNT with the laser frequency set to 1.96 eV near the E_22_ band, as well. Both spectra are normalized with respect to the G mode intensity. Panel a is reproduced from ref (2). Copyright 2022 Science Publishing Group. Panel b is reproduced from ref (29). Copyright 2022 Springer Nature Publishing Group.

In this Letter, we discuss theoretical simulations performed on the (9,1) SWCNT chirality to elucidate effects on simulated near-resonant Raman spectroscopy after functionalization using a perturbation of the electronic structure of the material. In this way, we identify vibrational modes that can be interpreted as those stemming from the defect by varying the frequency of the external Raman laser near the E_11_* transition.

To rationalize the experimental findings, in which a variety of SWCNT chiralities were examined, two (9,1) SWCNT models were chosen as the focus of our computational study. We expect our results to be easily transferable between chiralities, noting that the most important difference will be in the E_11_* transition energy region, which is well-known from previous reports.^24^ This allows us to choose the (9,1) SWCNT as our test case for the numerically intensive resonant Raman simulations. Panels a and b of Figure 2 show the atomistic models of pristine and *ortho*(++) aryl/aryl sp^3^-defected SWCNTs, respectively (see Methods for an explanation of defect and capping schemes). Figure 2c depicts the *ortho*(++) configuration (red) for the (9,1) chirality. The calculated absorption spectra for the two SWCNT models are shown in panels d and e of Figure 2, which will be used later to motivate choices of perturbative Raman calculations. For the pristine case, one can find the corresponding major transitions seen in experiment E_11_, E_22_, and E_33_ near 1.45, 1.80, and 2.15 eV, respectively. The transition energies match very well the reported experimental values.^68,69^ For the defected case, the energies for each of the pristine transitions are only weakly modified, but the intensity of the E_11_ transition becomes much reduced due to the presence of the defect. The E_11_* transition appears at 1.25 eV with a red-shift from the pristine E_11_ of ∼200 meV, which is consistent with previous similar calculations.^24^ In the simulated spectra, the optical intensity of the various Van Hove singularities (E_11_, E_22_, E_33_, etc.) is known to increase with energy in finite-length SWCNT models,^70^ contrary to experiment in which the E_11_ transition is usually the strongest in pristine samples. Additionally, in the defected case, the defect-associated E_11_* exciton borrows oscillator strength from the pristine E_11_ transition in the finite-sized models, effectively reducing the pristine intensity. Further, we note that changing the defect configuration will modify the resulting defect-associated excitonic transition E_11_* (∼100 meV).^24,30^However, we do not expect the conclusions of this Letter to be altered by the energetic location of this transition.

**Figure 2:**
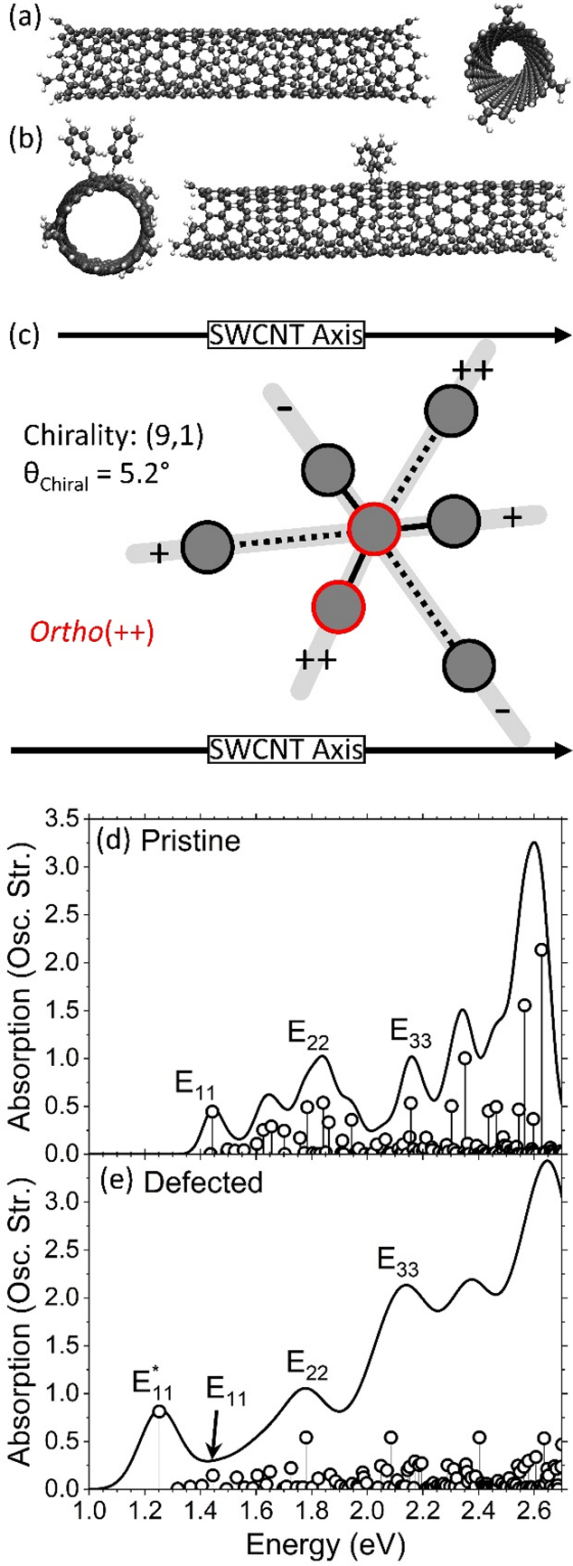
(a and b) Pristine and *ortho*(++) aryl/aryl defected (9,1) SWCNT structures used in this work, respectively. (c) Definition of the *ortho*(++) functionalization configuration (red) on the SWCNT surface with respect to the other five possible (two *ortho* and three *para*) local arrangements reported in previous works.^4,24,30^ Simulated absorption spectra for the (d) pristine and (e) defected (9,1) SWCNTs. The excitation energies were broadened by a Gaussian distribution with widths of 0.05 and 0.1 eV for the pristine and defected cases, respectively, for the sake of visual clarity.

Figure 3 shows the off-resonant Raman spectra for the pristine (black) and defected (red) SWCNT models. The G and D bands are present in the pristine and defected SWCNTs because of the finite SWCNT used for the modeling. The edges activate D-like Raman modes. However, upon functionalization of the SWCNT, the intensity of he D band increases sharply, indicating that the defect mode dominates the intensity of this band. Note that the intensity of the G band is also increased as a result of functionalization (see Figure S1 for normalized results with respect to the G band peak which is commonly done in experiment). The inset shows the small perturbations to the low-frequency Raman modes induced by the defect. The intensities of the modes around 500 and 600 cm^–1^ are appreciably increased compared to those of the pristine system, but the total intensity is largely dominated by the G and D bands. In comparison to Figure 1, the calculated off-resonant Raman spectra do not showcase the same interesting features recently explored in experiment. In this case, only the features present in the pristine SWCNT are modified (i.e., increased in intensity).

**Figure 3:**
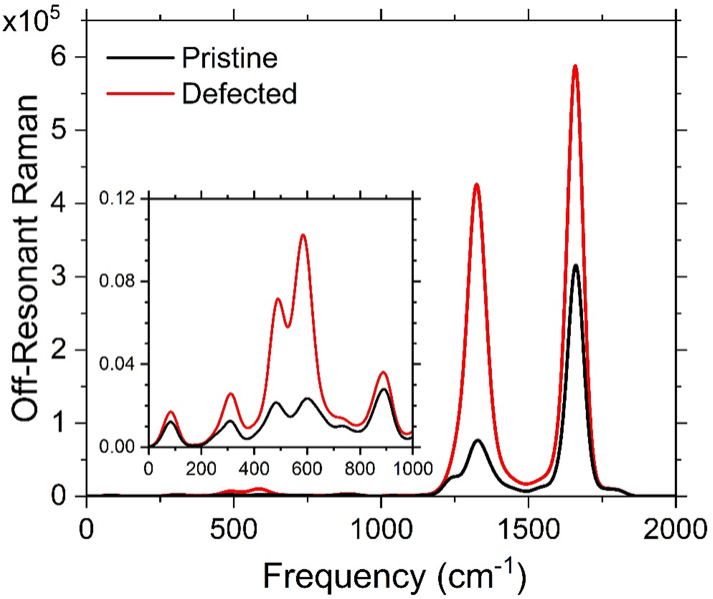
Computed off-resonant Raman spectra for the pristine (black) and defected (red) SWCNT species. The inset shows a close-up of the 0–1000 cm^–1^ region.

To gain a more experimentally relevant understanding of the Raman intensities, we now examine simulated resonance Raman spectra utilizing the coupled perturbed Hartree–Fock (CPHF) approach (see Methods and the Supporting Information). This technique can directly examine effects originating from the resonant coupling of the laser field to electronic transitions, which was done in experiment by coupling to the E_22_ transition of the SWCNTs.^2,29^ Compared to the off-resonant Raman spectral modeling (Figure 3), the CPHF Raman spectral calculations are very expensive. We start with analysis of the Raman spectra near the E_11_* transition of 1.25 eV at varying perturbative frequencies. Panels a and b of Figure 4 show pristine Raman spectra at five unique perturbative energies (*E*_CPHF_): 1.05, 1.15, 1.20, 1.30, and 1.35 eV. Note that the CPHF technique cannot produce spectra exactly at or closer to the E_11_* transition energy due to numerically diverging coupling, which produces non-real-valued results in the computational software, and we do not expect the perturbative CPHF approach to perform well for these truly on-resonant cases in which a higher-level method, for example sum-over-states polarizabilities, would be required.^71^

**Figure 4:**
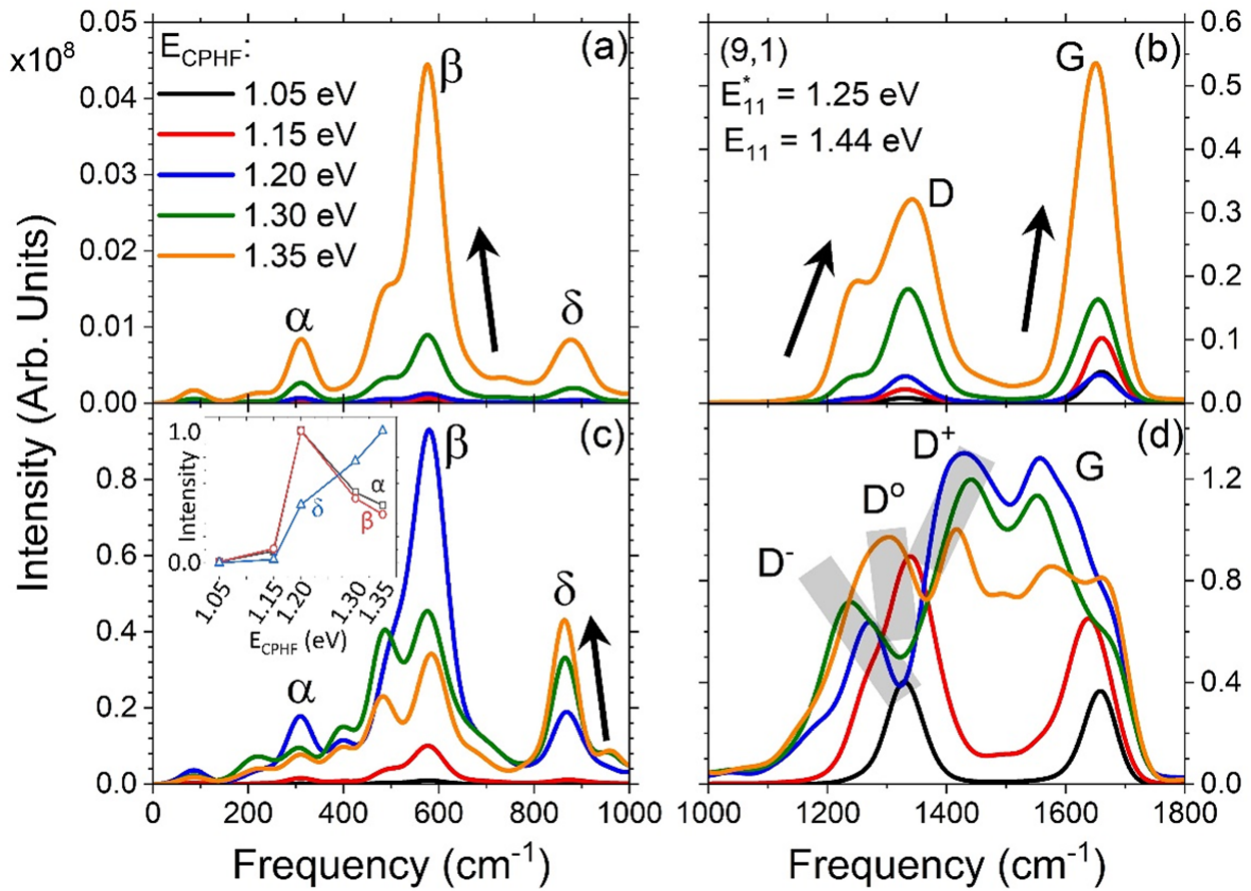
Computed near-resonant Raman spectra at varying perturbative laser energies (colors) for the (a and b) pristine and (c and d) defected SWCNT models. The spectra are split into (a and c) low-frequency and (b and d) high-frequency regions for the sake of visual clarity. Note the variation in vertical scaling among the four panels. The perturbation energies are all near the region of the computed E_11_* transition of 1.25 eV. The arrows indicate monotonically varying trends with respect to the increasing perturbative energy where applicable. The inset in panel c showcases the strong resonance effects in modes α and β found in panel c, where each intensity curve is normalized with its own maximum intensity. The same data for pristine bands are shown in Figure S3. The light gray boxes in panel d showcase the three defect-associated mode trends labeled as D^–^, D°, and D^+^.

Panels c and d of Figure 4 show the same calculations but with the *ortho*(++) aryl/aryl defected SWCNT. The spectra in each case are split between the low-frequency (panels a and c) and high-frequency (panels b and d) ranges for the sake of visual clarity. The intensity-normalized version of this figure can be found in Figure S2. The high-frequency pristine spectrum (Figure 4b) at a far-detuned perturbation frequency (*E*_CPHF_ = 1.05 eV) shows vanishing intensity everywhere except a single peak at 1650 cm^–1^ near the G band of the computed off-resonant Raman spectrum for the pristine system (Figure 3). At less detuned CPHF frequencies, we see a systematic increase in the intensity of the G band as well as an increase in the intensity of the D band around 1350 cm^–1^. We also note the presence of a lower-energy sideband to the D mode appearing at 1250 cm^–1^, which is also present in the off-resonant case (Figure 3). The low-energy spectrum of the pristine system (Figure 4a) showcases a monotonic (denoted as a black arrow) increase of three main bands at roughly 300, 600, and 900 cm^–1^, denoted as α, β, and δ, respectively. Note the vertical scaling is an order of magnitude smaller than that for the high-frequency region. We will see below that the modes near 900 cm^–1^ (δ) can be attributed partially to activated modes residing in the central portion of the SWCNT as well as partially (to a smaller degree) attributed to activated edge modes of the system that are weakly coupled to the delocalized excitations in the pristine system (Figure 2c). Some of these are optically dark but have non-zero oscillator strengths and may have strong overlap with the edge modes, which then contribute defect-like changes in their intensity. Changes to the IFM region with varying incident LASER frequency for a variety of SWCNT chiralities were also shown, experimentally, in ref (59). Our results exhibit very similar activity in the 200–900 cm^–1^ region with a strong dependence on the frequency; however, the simulated intensity in pristine SWCNT models at these low incident frequencies (far negatively detuned from the usual E_22_ excitation) is much reduced compared to those reported in ref (59). We will see below that higher incident frequencies give rise to intense and broader Raman signals comparable to those of the G and D mode signals.

Turning to the defect-associated spectra for the low-frequency region (Figure 4c), we find that similar modes become activated as seen in the pristine system. However, the intensities of the modes are, in general, much larger, and two of the three main modes at 300 (α) and 600 (β) cm^–1^ undergo non-monotonic intensity changes (where the black arrows were removed in comparison to Figure 4a). This strongly contrasts the pristine case due to the introduction of the defect E_11_* transition at 1.25 eV. The intensity of the mode near 900 cm^–1^ (δ) is monotonically increasing with CPHF energy, indicating that this mode strongly couples to the intermediate-energy dark exciton states with non-zero edge character, which will be revisited below. The intensity of the mode near 600 cm^–1^ (β) reaches a maximum at an *E*_CPHF_ of 1.20 eV (blue) with a very weak lower-energy sideband at 450 cm^–1^. The higher-frequency CPHF calculations (green, *E*_CPHF_ = 1.30 eV; orange, *E*_CPHF_ = 1.35 eV) show decreasing intensity with increasing energetic detuning from the E_11_* transition. Similarly, the farthest negatively detuned calculation at an *E*_CPHF_ of 1.05 eV (black) has vanishing intensity, and the second-most negatively detuned (red, *E*_CPHF_ = 1.15 eV) shows only the main band at 600 cm^–1^ (β). The sidebands around 450 cm^–1^ are most dominant at *E*_CPHF_ values of 1.30 and 1.35 eV. The mode at 300 cm^–1^ (α) also shows the same trends as the mode at 600 cm^–1^ (β) but with a lower intensity. These two modes at 300 (α) and 600 (β) cm^–1^ originate from the sp^3^ defect located at the center of the SWCNT and demonstrate that these intermediate energy frequencies (200–700 cm^–1^) are enhanced in the experimental resonant Raman spectra due to the sp^3^ lattice defect, again with the exception of the mode near 900 cm^–1^ (δ). This band, however, as we will see below, exhibits competing modes at similar frequencies localized to the center of the SWCNT that dominate the intensity as well as edge-localized modes.

Figure 4d shows the high-frequency CPHF Raman spectrum for the defected case. For the highly detuned, low-frequency calculation at *E*_CPHF_ = 1.05 eV (black) and the next nearest point at *E*_CPHF_ = 1.15 eV (red), the off-resonant D and G bands are recovered. Interestingly, the D band intensity dominates over the G band intensity. For the *E*_CPHF_ = 1.20, 1.30, and 1.35 eV calculations, two new main modes are introduced between the D and G band peaks near 1450 and 1550 cm^–1^. This parallels the complete disappearance of the D and G band peaks for the 1.20 and 1.30 eV cases. Moving from an *E*_CPHF_ of 1.05 to 1.30 eV, we see shifts in both the frequency and intensity of a new sideband to the D band peak, denoted as D^–^, starting near 1300 cm^–1^ for *E*_CPHF_ = 1.05 eV and ending near 1200 cm^–1^ for *E*_CPHF_ = 1.30 eV. The *E*_CPHF_ = 1.35 eV spectrum is rather unique compared to the others, as it reflects coupling to all nearby excitons, notably the E_11_*, intermediate dark states, and E_11_ electronic transitions. This gives both the new intermediate (between D and G modes) and the D and G modes themselves modified features distinctly different from those in the off-resonant Raman spectra. The gray boxes are aids for the eye in understanding the trends in the D mode modification of the new bands denoted as D^–^, D°, and D^+^. These observations contrast with the changes seen in the pristine case. In fact, the resonance effects of the D mode reported in ref (60) agree extremely well with our simulated spectra. Both results showcase multiple D mode peaks at higher frequencies (D^–^, D°, and D^+^ in our notation) but only a single intensity peak (D°) at a low frequency with roughly linear scaling of the intensity with an increasing incident frequency.

To better understand the character of the modes activated by resonance effects, we seek a method for understanding and characterizing the modes that contribute largely to the Raman spectroscopy shown in Figure 4. In this spirit, one can decompose the normal modes on the basis of their contributions at each point (i.e., slice) along the SWCNT main axis. Figure S4 provides a visualization of the position-resolved probability amplitudes for each normal mode (eq S15 in the Supporting Information) for (a) pristine and (b) defected SWCNT models. Various types of mode localizations can be seen: (I) edge-localized, (II) defect (center)-localized, and (III) delocalized. The data presented in Figure S4 can be reformulated in various ways, and we have included such depictions in Figures S5–S8 to aid the interested reader in obtaining a more well-rounded understanding of the analysis performed in this work and how it aids in the characterization of the normal modes. Figure S5 shows an analogous depiction of Figure S4 in the form of a two-dimensional heat map. In addition, taking these discrete probability distributions and convolving with a finite-width Gaussian function in the frequency domain (eq S17 in the Supporting Information), one obtains an amplitude density map as shown in panels a and b of Figure 5. The pristine (Figure 5a) and defected (Figure 5b) SWCNT maps show large differences in the central region of the SWCNT axis, indicating the presence of the defect-localized modes. A three-dimensional contour map is shown for the sake of visual clarity in Figure S6 using the same data as in Figure 5b. Finally, the difference amplitude density between the pristine and defected density maps is shown in Figure S7, showcasing the contributions from the defect on each mode.

**Figure 5:**
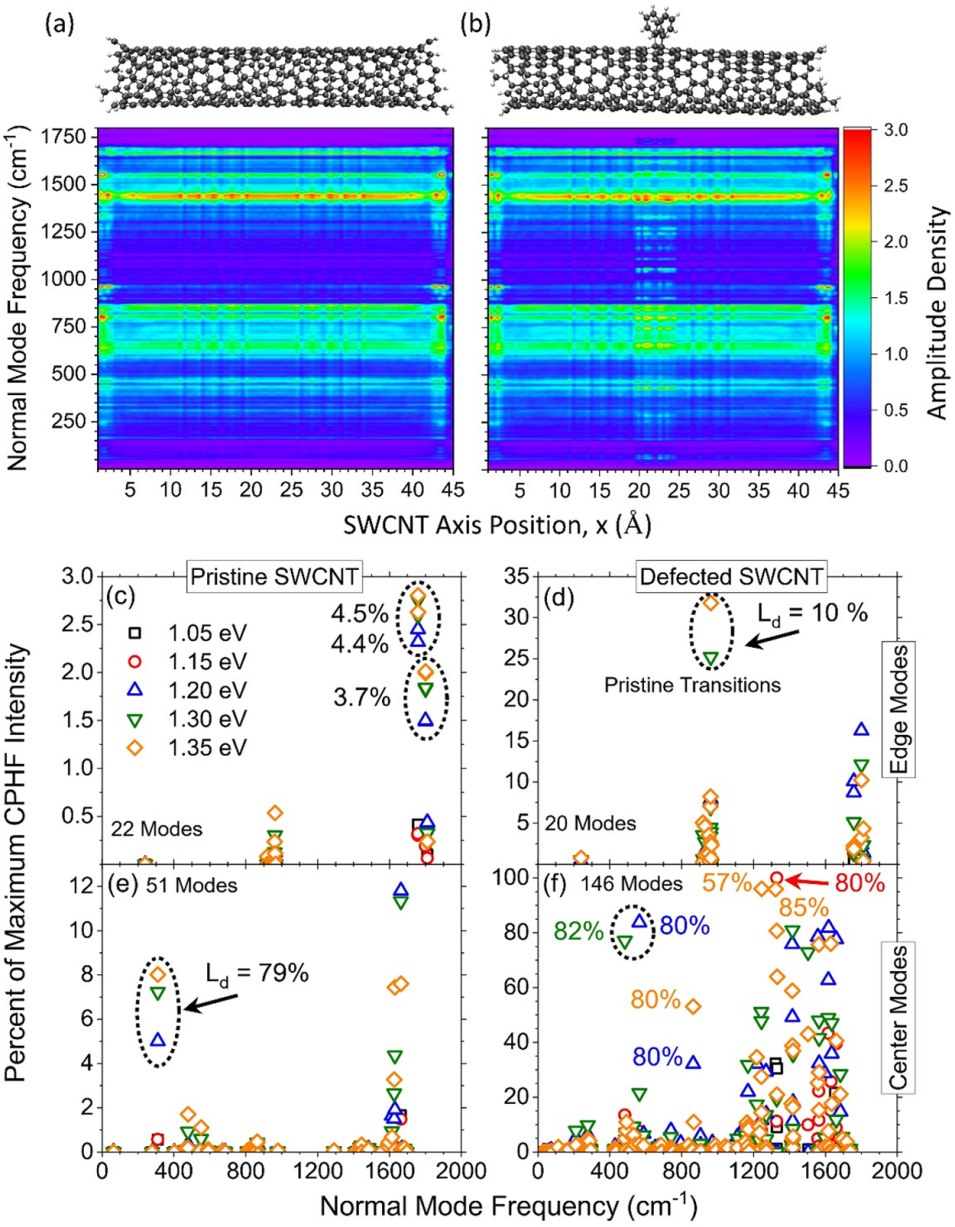
(a and b) Amplitude density map of the normal modes plotted as a function of SWCNT axis position, *x*, and frequency for the (a) pristine and (b) defected SWCNT models. The probability amplitudes are convolved with a Gaussian function of width σ of 5 cm^–1^. (c–f) Percent CPHF maximum intensity of normal modes localized within (c and d) 5 Å of the edges by 35% and (e and f) to the center 10 Å by 30% of the (c and e) pristine and (d and f) defected SWCNT models for all CPHF perturbation energies. The number of modes that fall inside the criteria is shown in each panel. Note the differences in vertical scales among all four panels. In all panels, some points are labeled by their *L*_d_ (percent) value as the percent normal mode extent along the SWCNT axis.

Moving to more quantitative descriptions of the mode character, we can compute an effective localization parameter, also termed the inverse participation ratio,^30,72−74^*L*_d_, for each normal mode (eq S18 in the Supporting Information), and these data are overlaid upon the same data as presented in Figure 4 and can be found in Figure S8. The localization of the normal modes ranges widely from near 10% (indicating a spatial localization to 10% of the SWCNT axis) to near 90% (nearly delocalized case across the entire SWCNT) for the pristine (Figure S8a) and defected (Figure S8b) SWCNT models. The *L*_d_ parameter provides a quantitative measure of the normal mode extent along the SWCNT axis and will be used to help understand and partition the normal modes into quantifiable classes of SWCNT modes.

From the spatially resolved probabilities (eq S16 in the Supporting Information), we further define the first two classes of modes, I and II from above, as the modes with (I) >35% edge character and (II) >30% center character, respectively (see the Supporting Information for additional details). The intensity of these modes is then plotted for each external laser frequency as the percent of the maximum intensity for each frequency. Panels c–f of Figure 5 showcase the results for edge localization (Figure 5c,d) and center localization (Figure 5e,f) for the pristine (Figure 5c,e) and defected (Figure 5d,f) SWCNT models. Notably, with this criterion, there are 22 and 20 modes with edge character for pristine and defected cases, respectively. The intensity of these modes in the pristine system is <3%, while in the defected model, some modes are present around 30% (∼900 cm^–1^) with the higher laser frequencies (*E*_CPHF_ = 1.30 and 1.35 eV). This is evidence that stronger coupling to the more delocalized excitonic states activates the edge modes that appear as mostly dark excitonic states in the absorption spectrum for the defected SWCNT (Figure 2e). These states with edge character exhibit a small feature in the near-resonant Raman spectrum (Figure 4a,c) for the pristine and defected cases that becomes more intense with an increase in CPHF frequency. These mostly dark excitonic states mediate the coupling to activate these edge modes (see Figure S9 for the real-space projected transition density isosurfaces of the five lowest excitonic transitions in the defected model). The edge-localized mode near 1800 cm^–1^ should exhibit some features in Figure 4b, but the Gaussian broadening eliminates them due to the high density of features at lower frequencies. As such, these are not resolved in the spectra for a Gaussian width σ of 25 cm^–1^.

The center-localized modes in the pristine SWCNT model (Figure 5e) exhibit two main features at ∼300 and 1650 cm^–1^ with ≤12% CPHF Raman intensity compared to the maximum. The defected model (Figure 5f) showcases 3 times as many center-character modes with additional features at 500, 600, and 850 cm^–1^ with a band of activations between frequencies of 1200 and 1600 cm^–1^ with CPHF Raman intensity up to 100% compared to the maximum for the modes in the 1200–1600 cm^–1^ range for all external laser frequencies, except for the strongly detuned *E*_CPHF_ = 1.05 eV frequency. The overlapping-in-frequency modes that are edge- and center-localized near 300, 500, and 900 cm^–1^ become washed out once the defect states are activated. This analysis has shown that although the edge modes can be activated through resonant coupling, the center-localized, defect-induced modes dominate the spectrum for all choices of external laser frequency and therefore weakly participating edge modes largely do not affect the conclusions reached above. Thus, we have proved the validity of our model for analyzing the modes activated by resonant Raman spectroscopy in experiment.

For the sake of completeness, Figure S10 shows pre-resonance Raman spectra for two additional CPHF energies: 1.45 eV (panel a) and 1.75 eV (panel b). These energies are near the computed E_11_ (1.44 eV) and E_22_ (1.78 eV) excited state transitions of the system. In both cases, the systems exhibit a largely delocalized Raman intensity distribution. Notably, the D and G bands from the off-resonance Raman spectrum have completely disappeared, and a new peak at 1500 cm^–1^ is present in the pristine and defected cases at both perturbation energies. The low-frequency range exhibits a broad set of peaks centered around 600 cm^–1^ as shown in Figure 4c; however, nearly all adjacent modes have now been enhanced through coupling to the electronic transitions of the pristine tube, now with a much stronger Raman signal. This result supports findings from experiments performed with incident frequencies near the bright pristine-associated excitons.^2,29,42−53,59^

Figure S11 displays an alternative depiction of the intensity through an intensity-difference function (Δ*I* = *I*^DEF^ – *I*^PRISTINE^). These features stem from the coupling of many modes along the SWCNT axis to the bright E_11_ and E_22_ transition with strong oscillator strengths, as well as to all other semibright excitations that are nearby in energy. This effectively washes out the small details pointed out above while coupling to the weaker transition with a small number of nearby-in-energy excitons (i.e., low density of states). However, there are a couple important facets to these high-frequency calculations. Unexpectedly, the CPHF method results in a decrease in intensity near the G mode frequencies (∼1500 cm^–1^) for the E_11_ coupling (Figure S10a), where usually the CPHF approach would yield an overwhelming increase in intensity when coupling to such a bright transition. This may be due to the reduction in oscillator strength of the E_11_ exciton after placement of the defect (Figure 2c,d). One important implication of this work is as follows. Modifying the external laser frequency results in drastic changes to the Raman spectra obtained through coupling to nearby bright and semibright electronic transitions. In the experiment, often this tunability remains unexplored (i.e., often only coupling to the E_22_ transition is investigated). However, this additional control may give rise to different spectra for the same molecular system even at slightly different incident light frequencies. In other words, coupling only to the E_22_ bright transition may not be providing the entire story, as additional electronic frequencies may activate new Raman modes and provide valuable information about the structure of sp^3^ defects in SWCNTs.

In summary, this Letter reports results of *ab initio* simulations elucidating recent experimental resonant Raman spectroscopy data in chemically functionalized SWCNT species. These experiments pointed to additional Raman modes becoming activated after functionalization in multiple SWCNT chiralities. Our simulations showcase similar new features in near-resonant Raman spectra that have not been reported or explained before with atomistic simulations to the best of our knowledge. We attribute these new features to those vibrational modes that are strongly coupled to the defect-associated optical transition (E_11_*) due to the unique and pointed changes to these modes using varying incident laser frequencies near the E_11_* transition. We envision that this work will inspire more intricate future investigations, both experimental and theoretical, exploring the physical nature of these intermediate modes strongly coupled to the defect-associated excitonic transitions. Changing the topology of a defect configuration is known to modify the energetic position of the E_11_* transition energy. However, we expect that similar trends will persist for the Raman spectra given only a change in the local configuration of the defect. In contrast, changing the chemical functionalization may have a more dramatic effect due to the large reorganization of surface charge with strongly electronegative species.^7,30,31^ The exploration of chemical groups and configuration schemes will be the subject of future works. Additionally, we suggest that future studies focus on the effects of multiple localized defects as well as modifications due to the adduct’s chemical identity, which will lead to further tunability of nonradiative relaxation pathways that can be explored with resonant Raman spectroscopy analysis. With this new information, the community at large can devise ways to utilize these lower-energy vibrational modes to probe excitonic dynamics and devise new chemistries on SWCNT lattices.

## Methods

*Geometry and Electronic Structure*. The pristine (9,1) SWCNT was constructed with a single unit cell of length ∼4 nm, and its edges were capped with three methylene groups, which has been previously shown to recover the semi-infinite electronic structure of the SWCNTs.^24,70,75^ An aryl/aryl sp^3^ hybridization defect was formed on the surface by covalently attaching two aryl radicals to adjacent (i.e., *ortho*) carbons in a single ring in the *ortho*(++) configuration, which is defined as the *ortho* bond that lies approximately 65.2° from the SWCNT axis. These defect configurations have been well-defined in previous computational reports for this and other chirlaities.^24,25,30,31,35^

All geometries were optimized in the ground state electronic state at the density functional theory (DFT) level using the B3LYP functional and STO-3G basis. Similar functionals and basis sets have been previously shown to provide qualitatively accurate electronic structure energies and excitonic localization properties of these systems.^24,25,30,31,35^ The lowest 150 singlet excited states were computed for each system at the minimum of the ground state potential energy surface using linear-response time-dependent DFT (TD-DFT) using the same functional and basis. The real-space projected transition density was generated with the aid of the MultiWfn package.^77^

All ground, excited, and perturbative electronic structure calculations were performed using the Gaussian 2016 software package.^78^

## Supporting Information Available

Normalized Raman spectra and excitonic transition density isosurfaces, additional figures depicting the normal mode analysis performed, a general outline of the coupled perturbed Hartree–Fock equations, and a detailed explanation of the normal mode analysis (PDF). Additionally, four supporting movies are provided showcasing normal mode displacements for a few key vibrations:
Movie S1: Largest-intensity pristine (9,1) G-mode (MP4)
Movie S2: Largest-intensity defected (9,1) D-mode (MP4)
Movie S3: Defected (9,1) IFM region mode at Raman frequency = 578.2 cm^–1^ and *E*_CPHF_= 1.20 eV (MP4)
Movie S4: Defected (9,1) RBM (MP4)
