# Supplementary material for: Signatures of Chemical Dopants in Simulated Resonance Raman Spectroscopy of Carbon Nanotubes
Source: J Phys Chem Lett. 2023 Jan 30;14(5):1182–91. doi: 10.1021/acs.jpclett.2c03591 (PMC9923748; doi:10.1021/acs.jpclett.2c03591)
Supplement: Supplementary file 1 — jz2c03591_si_001.pdf [file jz2c03591_si_001.pdf]

Signatures of Chemical Dopants in Simulated Resonance Raman Spectroscopy of Carbon Nanotubes

Supporting Information

Braden M. Weight,[1,2] Ming Zheng,[3] and Sergei Tretiak[2*]

[1]*Department of Physics and Astronomy, University of Rochester, Rochester, NY 14627, U.S.A*

[2]*Center for Integrated Nanotechnologies, Center for Nonlinear Studies, and Theoretical Division Los Alamos National Laboratory, Los Alamos, NM 87545, U.S.A.*

[3]*Materials Science and Engineering Division, National Institute of Standards and Technology, Gaithersburg, Maryland 20899, U.S.A.*

Email: serg@lanl.gov

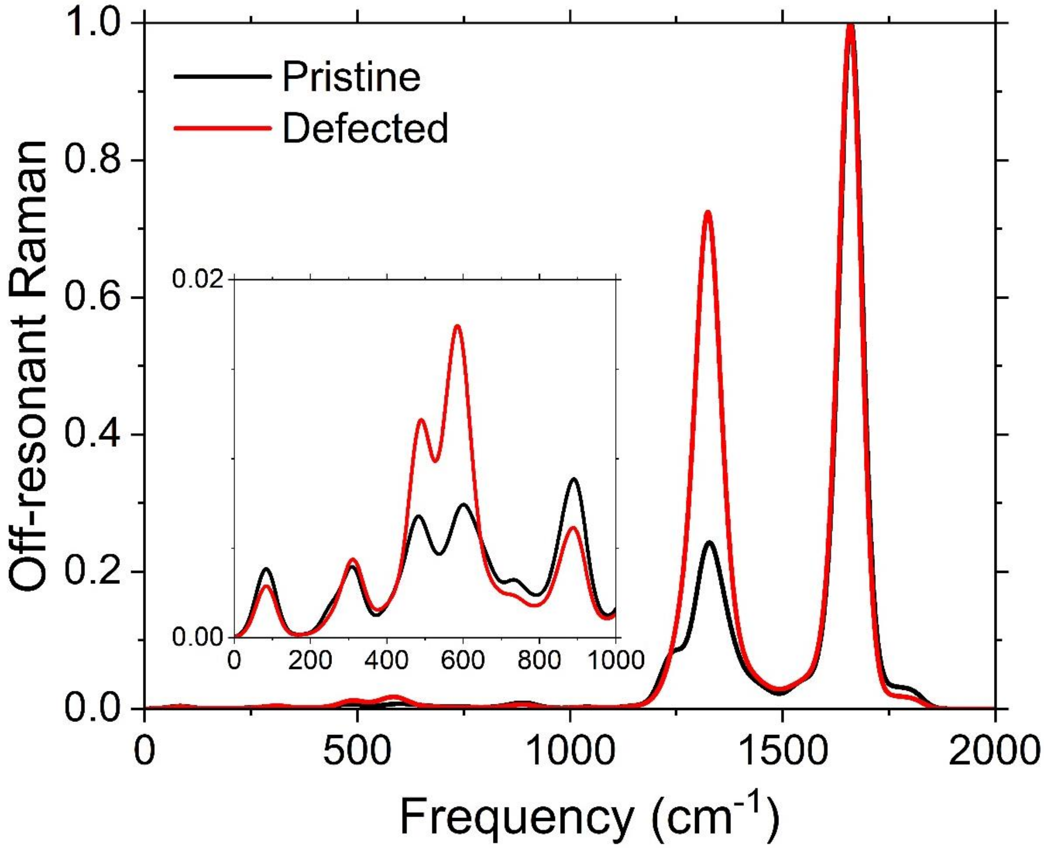

Figure S1: Normalized off-resonant Raman spectra for pristine and defected SWCNT systems. Normalization condition is defined as I → I / MAX(I).

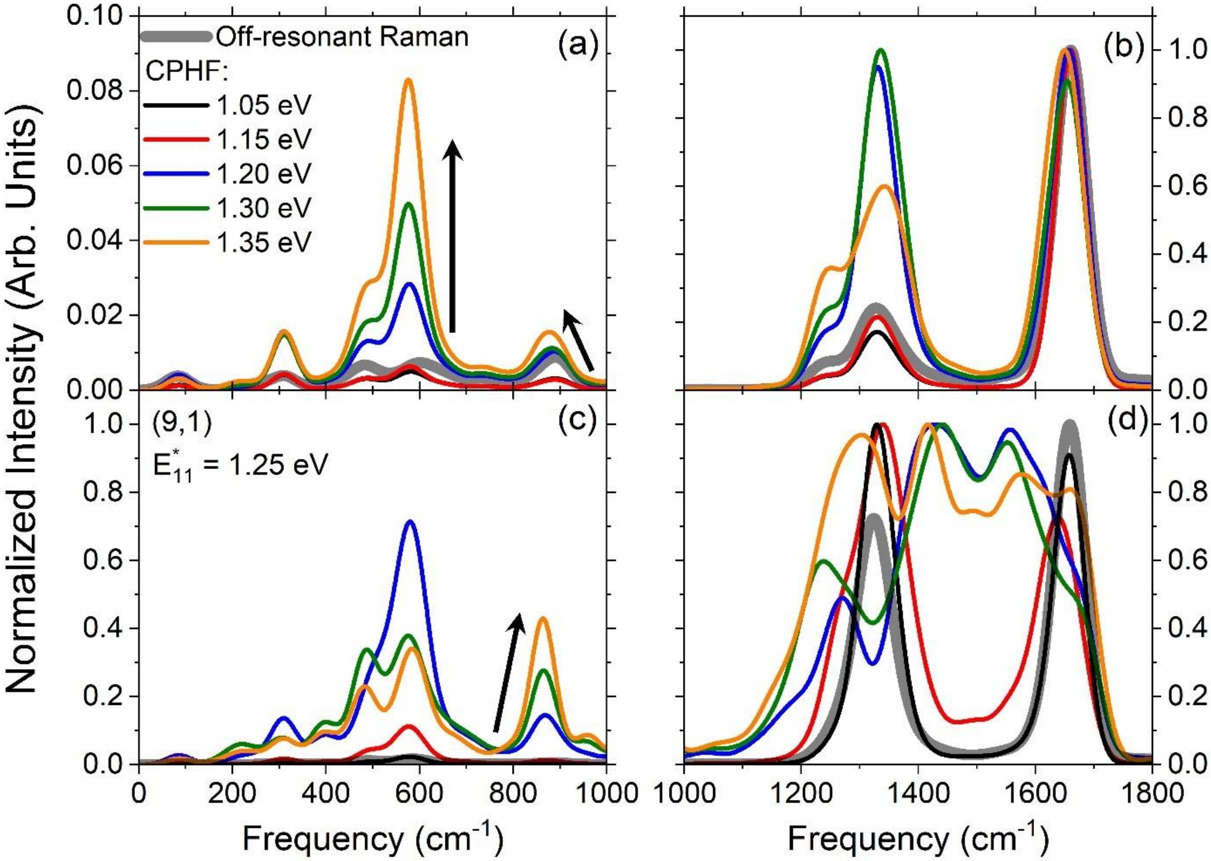

Figure S2: Normalized pre-resonant Raman spectra using the same as presented in Fig. 4. Normalization is defined as I → I / MAX(I). In experiment, usually, the normalization is usually defined as I → I / I("G-Mode"), but for our spectra, the definition of the G-Mode is not well-defined, especially for perturbation energies larger than 1.20 eV.

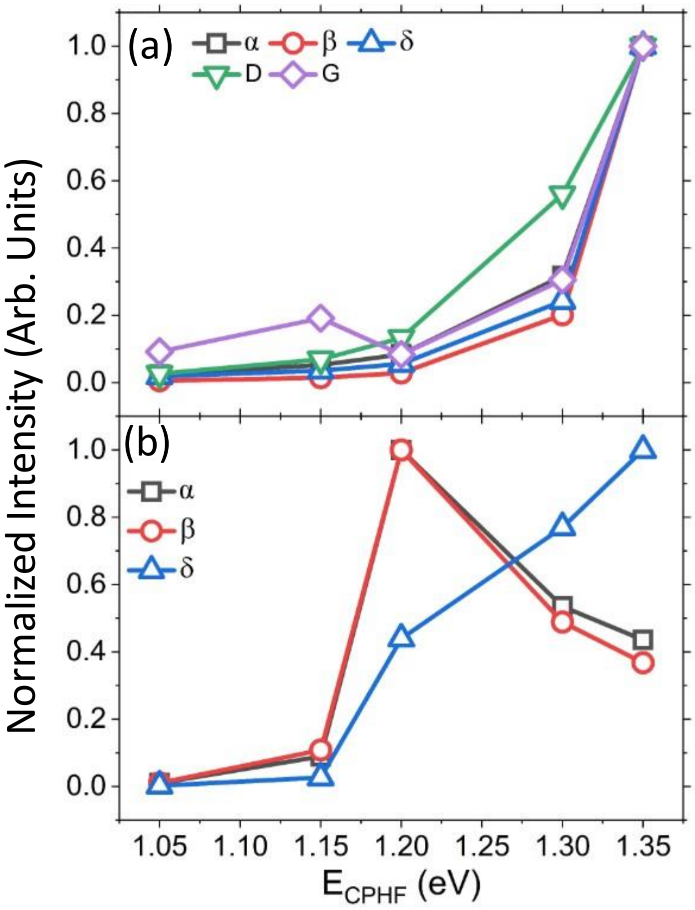

Figure S3: Raman intensity for a variety of major peaks that exhibit strong frequency dependence for the (a) pristine and (b) defected SWCNT models. The data is the same as that shown in Figure 4 in the Main Text with each curve normalized to its own maximum. The labels of the curves correspond to the labels shown in Figure 4.

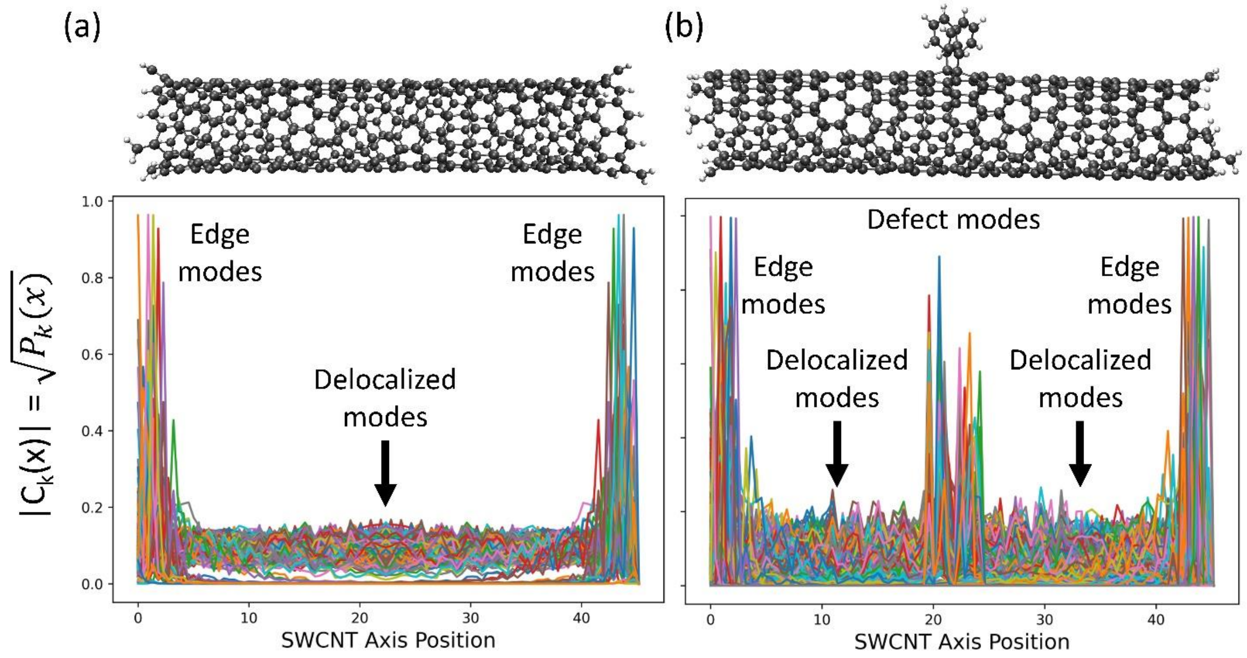

Figure S4: Position-resolved normal mode amplitudes for all normal modes *k* and with 100 spatial histogram bins along the SWCNT axis.

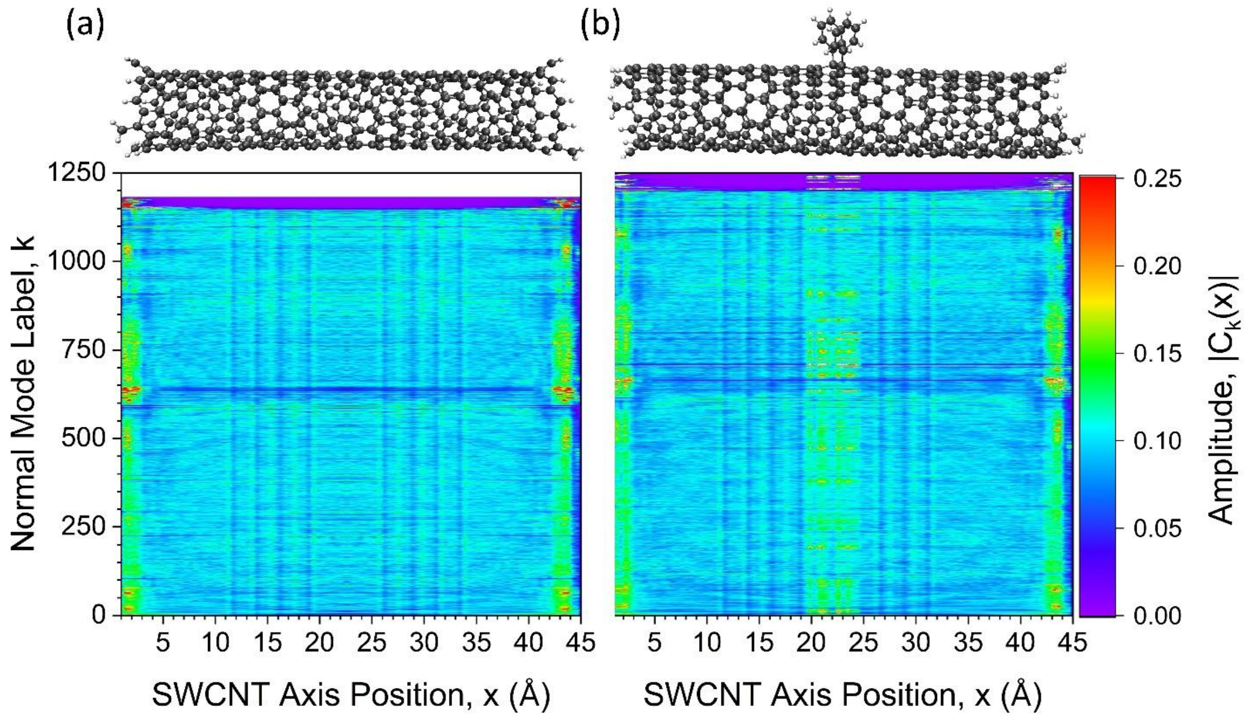

Figure S5: Position-resolved normal mode amplitudes as a function of the normal mode index and SWCNT axis position.

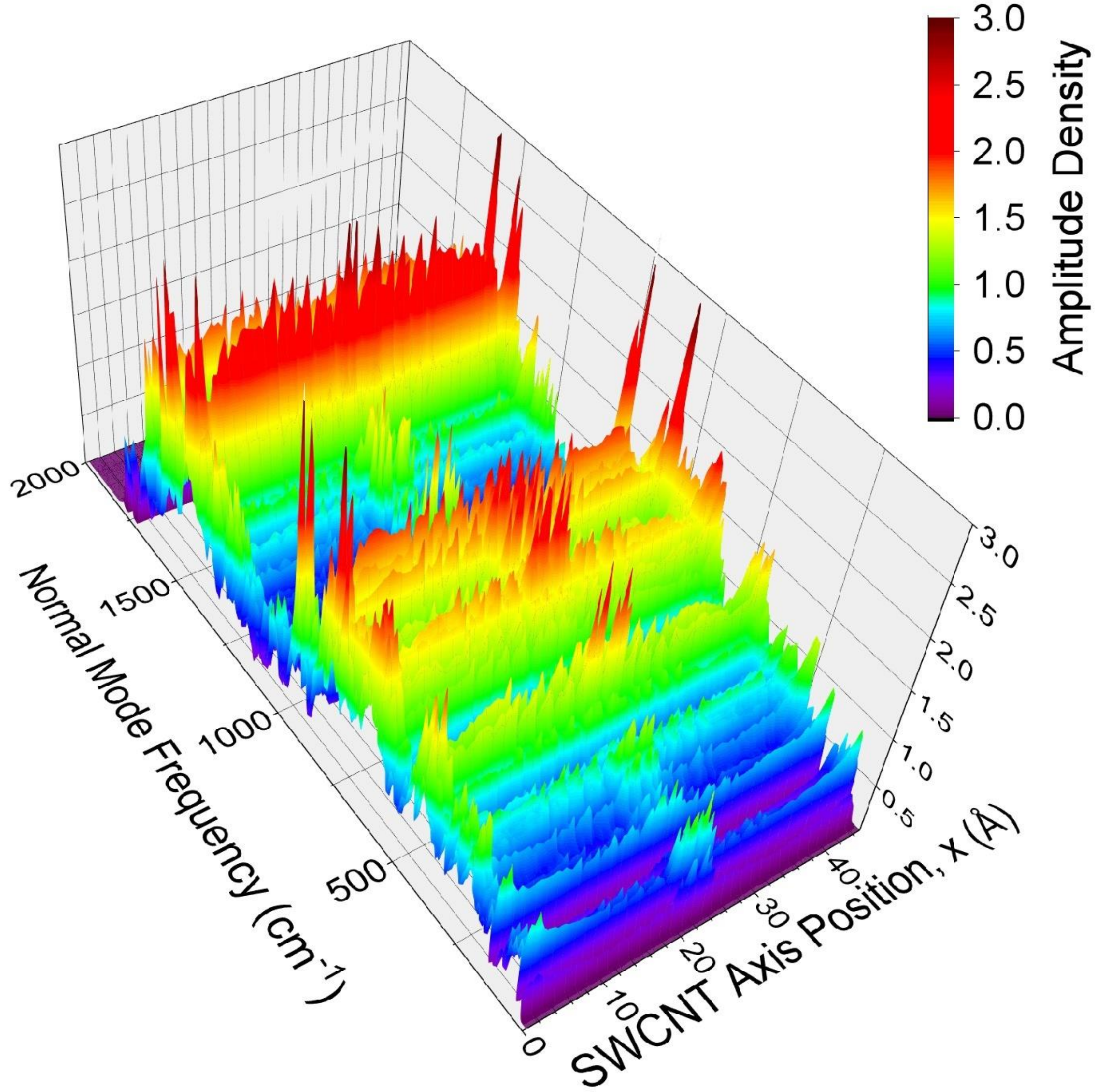

Figure S6: Position-resolved normal mode amplitude map as a function of normal mode frequency for the defected SWCNT. The normal modes were broadened with a Gaussian function of width σ = 5 cm^{-1}. This figure provides an alternative visualization of the same data shown in the main text Figure 5b and Supporting Figure 5b.

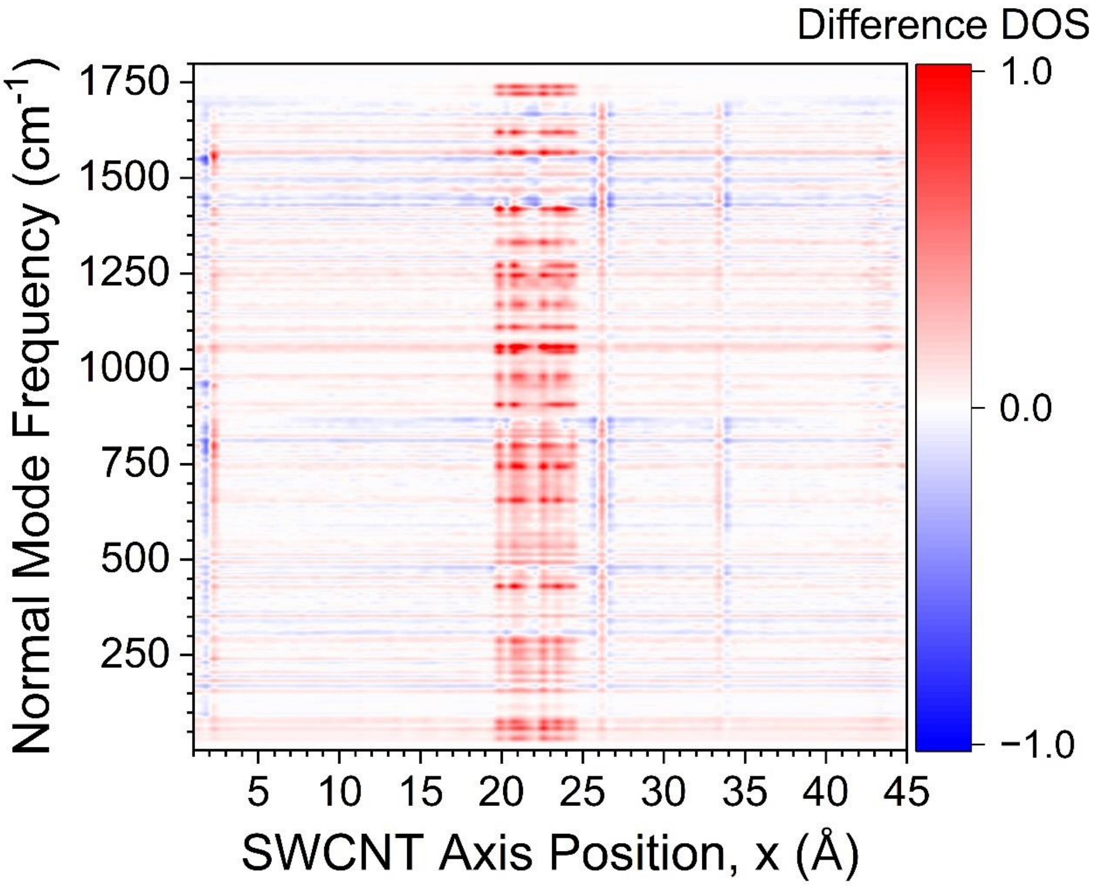

Figure S7: Position-resolved normal mode amplitude difference map as a function of normal mode frequency. The pristine density map was subtracted from the defected density map, with each density map having been broadened with a Gaussian function of width $\sigma = 5\ cm^{-1}$.

One can compute the different between the pristine and defected SWCNT amplitude density maps, which is presented in Fig. S8. Here, the defect modes are clearly visualized as a function of the normal mode frequency and SWCNT axis position with most effects stemming from the edges present in both the pristine and defected SWCNTs can be eliminated. Although, some edge effects are still seen, possibly due to the choice of discretization grid and can roughly be ignored since the adjacent positive and negative regions would exactly cancel for the peaks at the edges if binned together. The current spatial resolution is useful for examining the central portion of the SWCNT.

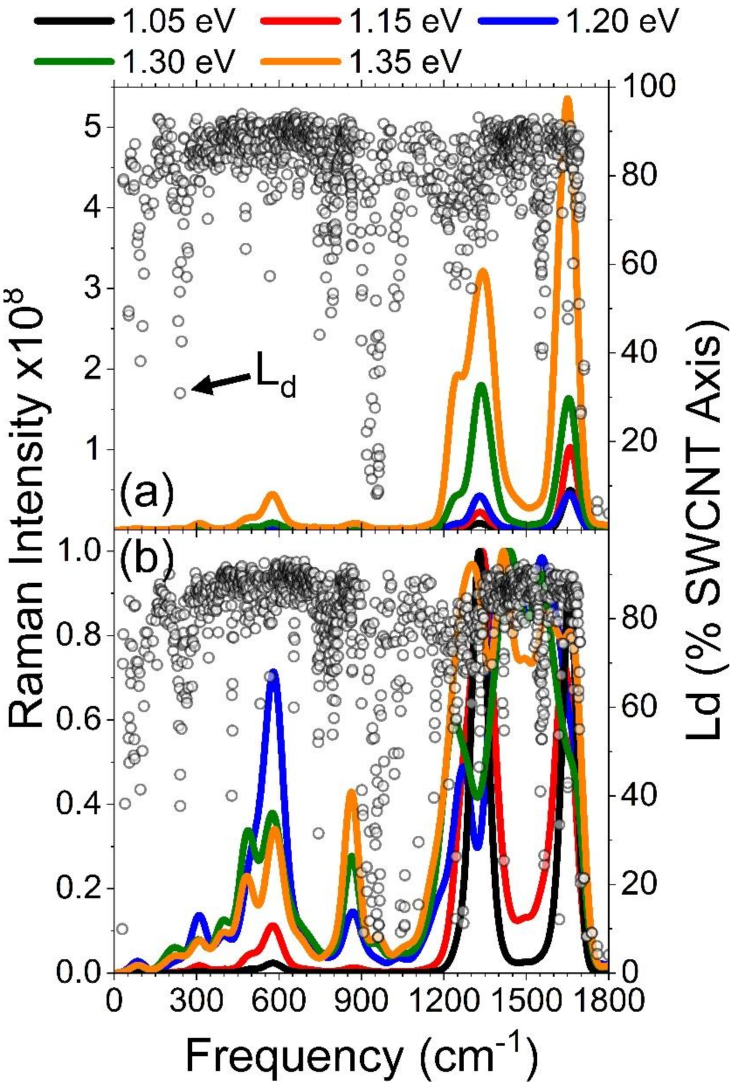

Figure S8: Pre-resonance Raman spectra for the (a) pristine and (b) defected SWCNT models replotted from Fig. 4 in the main text. The colors represent the CPHF perturbation frequency. The open black circles indicate the normal mode extent Ld parameter as a percent of the total SWCNT length. The mode near 900-950 cm^{-1} and 1800 cm^{-1} exhibit the most localized modes, having only ~10% or less extent along the SWCNT. The majority of features exhibit more than 40 % delocalization.

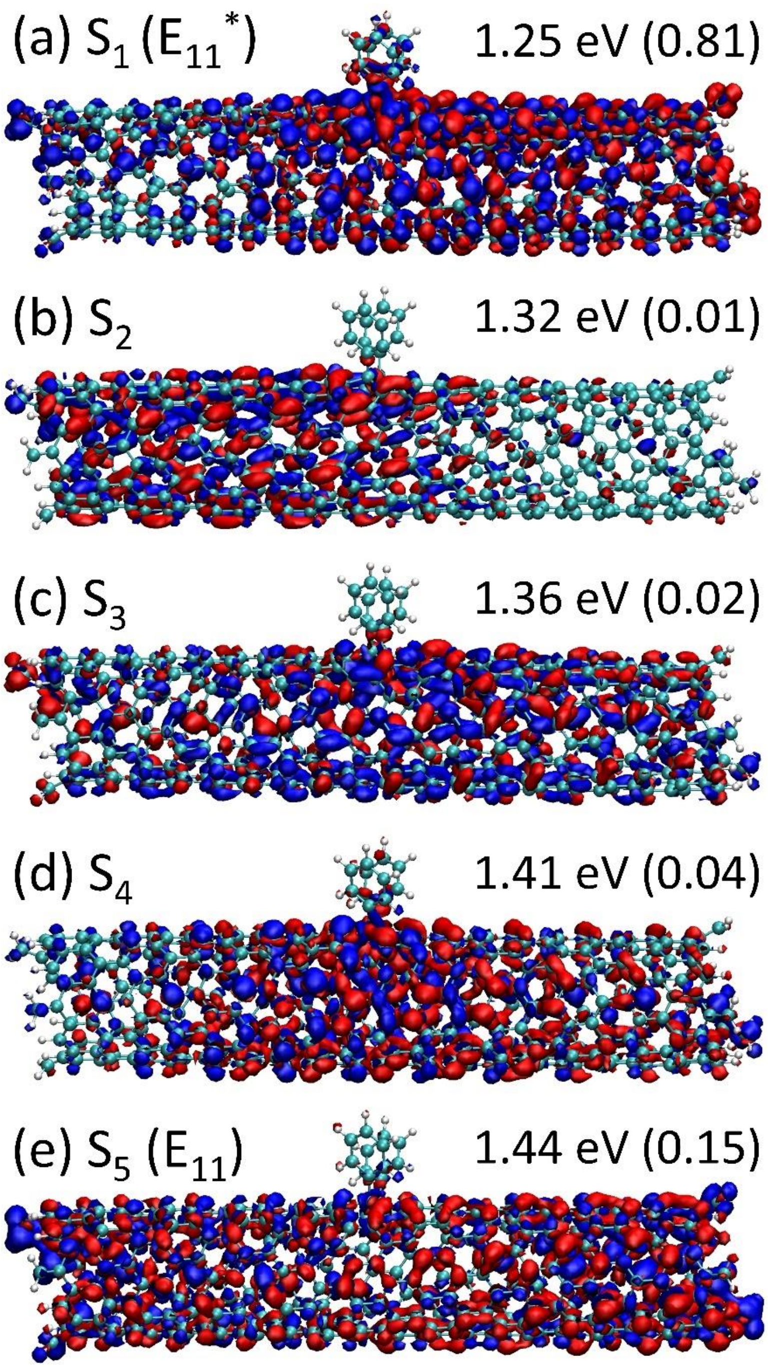

Figure S9: Real-space projected ground-to-excited transition density for the lowest five excitonic transitions. The transition energy and oscillator strength (in parenthesis) are shown for each transition. The S_1 is labeled as the E_{11}^* transition, and the S_5 is labeled as the E_{11} transition. The isosurface value is 0.0008.

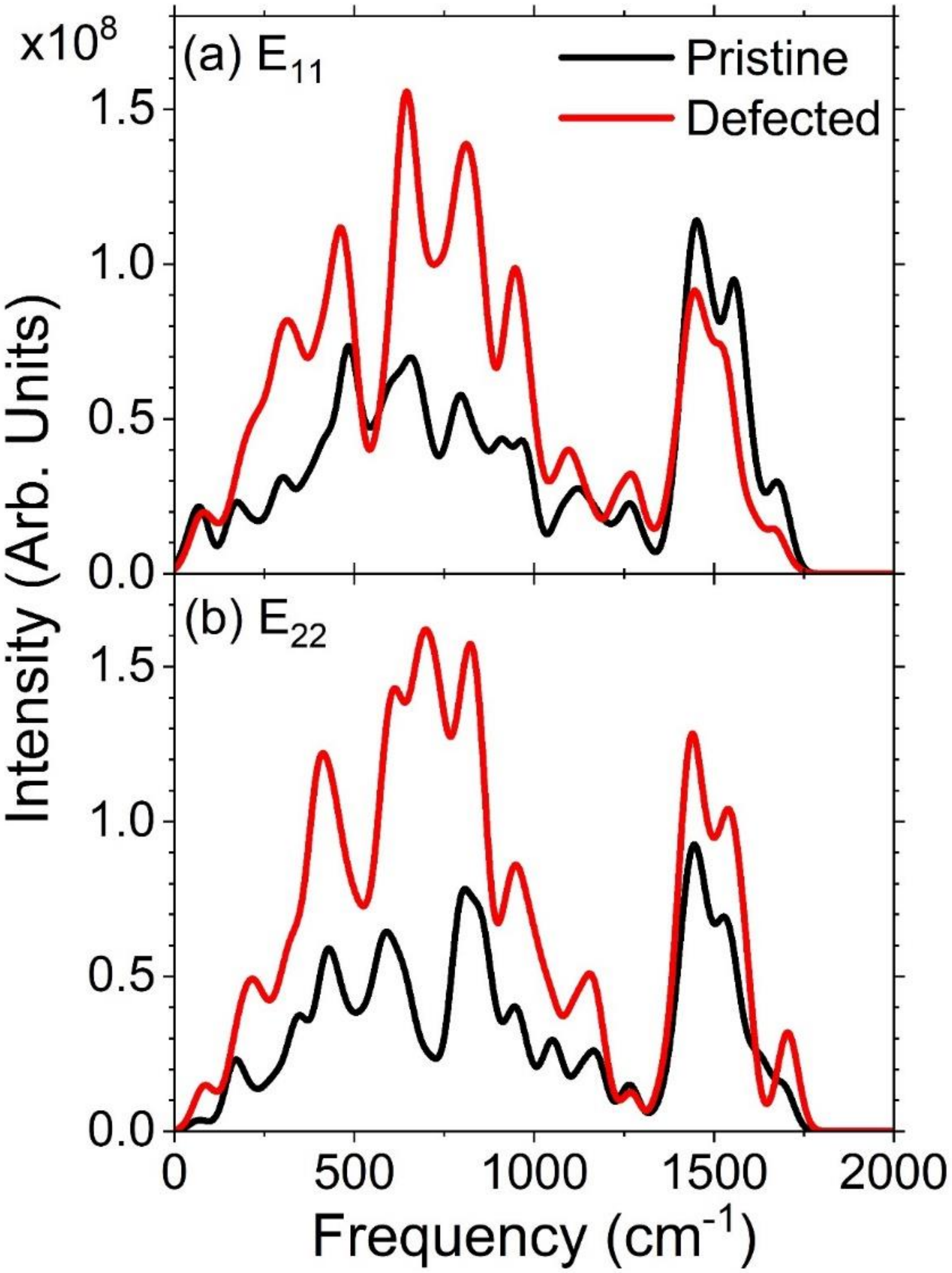

Fig. S10: Computed pre-resonance Coupled Perturbed Hartree-Fock (CPHF) Raman spectroscopy at two perturbative energies corresponding to (a) E_{11} (1.44 eV) with E_{CPHF} = 1.45 eV and (b) E_{22} (1.78 eV) with E_{CPHF} = 1.75 eV, respectively. The pristine (black) and defected (red) are shown together.

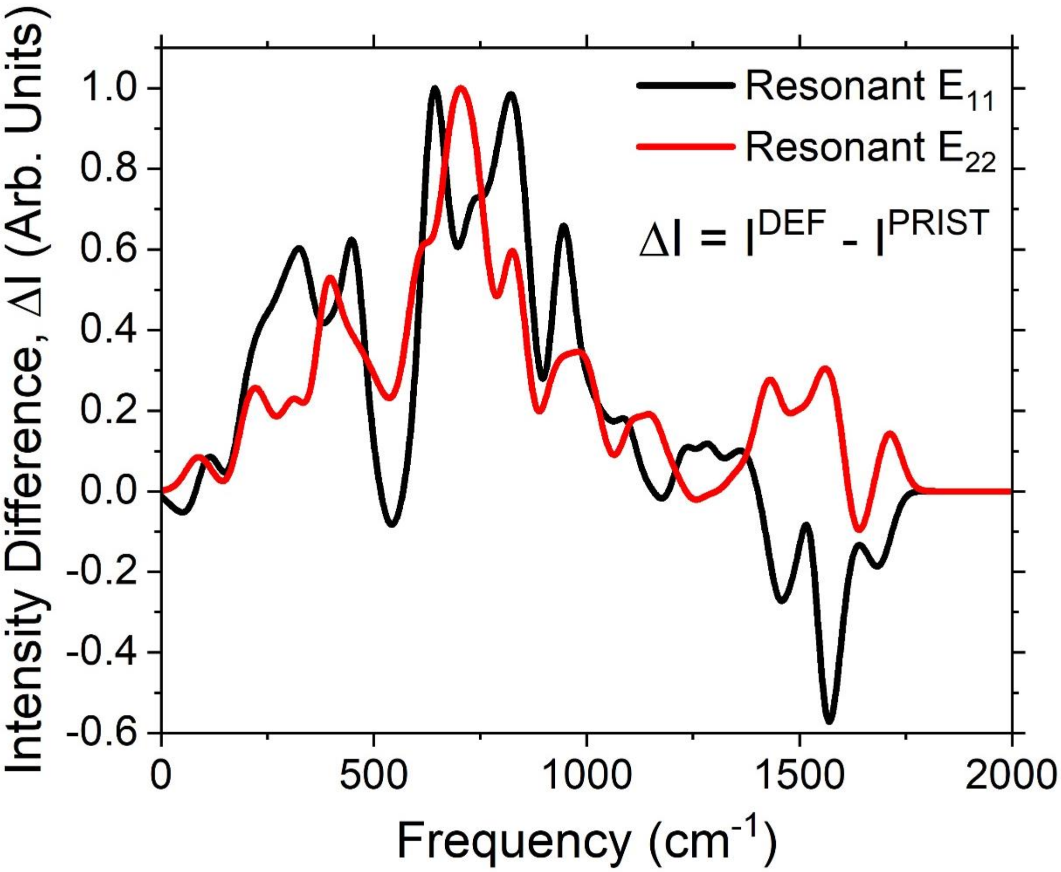

Figure S11: Intensity difference defined from the pre-resonant Raman spectra for the near E_{11} and E_{22} perturbation energies.

Additional Methodology

Coupled Perturbed Hartree-Fock

In order to model resonance effects of the vibrational system, the Coupled Perturbed Hartree-Fock (CPHF) equations were solved for the Kohn-Sham system at the ground state minimum geometries to obtain the near-resonance Raman spectra for various perturbation energies.[5–12] We will briefly outline the general framework behind the CPHF method following closely refs. [12–14].

In the presence of an oscillating external field $\boldsymbol{E}(t,\omega)$, one can perform a perturbative expansion around the electric field amplitude for the energy of the system,

$$\epsilon(\boldsymbol{E}) = \epsilon^{(0)} - \mu_a E^a - \frac{1}{2!}\alpha_{ab}E^aE^b - \frac{1}{3!}\beta_{abc}E^aE^bE^c - \frac{1}{4!}\gamma_{abcd}E^aE^bE^cE^d + O(\boldsymbol{E}^5) \quad (S1)$$

where ϵ^0 is the energy of the system without the presence of an external electric field, E^a is a component of electric field vector, μ_a, α_{ab}, β_{abc}, *and* γ_{abcd} are the tensor components of the dipole, polarizability, first hyperpolarizability, and second hyperpolarizability of the molecular system.[12] These quantities can be interpreted as molecular response functions dependent on the electric field $\boldsymbol{E}$. The total energy, as well as all future quantities, is assumed to be a sum of all components, *abcd*, following Einstein sum rules unless otherwise stated. Similarly, the total molecular polarization $P^a \sim -\partial\epsilon(\boldsymbol{E})/\partial E^a$ can be written as,

$$\mathrm{P^a}(\boldsymbol{E}) = \mu_a + \alpha_{ab}E^b + \frac{1}{2!}\beta_{abc}E^bE^c + \frac{1}{3!}\gamma_{abcd}E^bE^cE^d + O(\boldsymbol{E}^4). \quad (S2)$$

The polarization $\boldsymbol{P}(\boldsymbol{E})$ can be extracted from a wavefunction as,

$$P^a(\boldsymbol{E}) = \langle\Psi(\boldsymbol{E})|H^a(\boldsymbol{E})|\Psi(\boldsymbol{E})\rangle, \quad (S3)$$

where $H^a(\boldsymbol{E}) = \frac{\partial H(\boldsymbol{E})}{\partial E^a}$ with $H(E) = H_o + H'$. Here, H_o is the total Hamiltonian of the system in the absence of the external field, and $H' = -\boldsymbol{\mu}\cdot\boldsymbol{E}$**.** From here, one can defined the ground state density matrix in terms of the single slater determinant (for Hartree-Fock- or Kohn-Sham-like calculations) as,

$$\hat{\rho}(\boldsymbol{E}) = |\Psi(\boldsymbol{E})\rangle\langle\Psi(\boldsymbol{E})|, \quad (S4)$$

with matrix elements defined as $\rho_{jk} = C_k^*C_jn_{jk}$, $\{C_j\}$ molecular orbital coefficients, and $n_{jk} = 2\delta_{jk}$ for $j < N_{Occ.}$ and $n_{jk} = 0$ for $j > N_{Occ.}$ as the occupation matrix. Now, the molecular polarization can be written as,

$$P^a(\boldsymbol{E}) = -Tr\left[\widehat{H}^a\hat{\rho}(\boldsymbol{E})\right], \quad (S5)$$

where $H^a_{\mu\nu} = -e\langle\mu|\hat{x}^a|\nu\rangle$ is the dipole matrix in the atomic orbital basis $\{\mu\}$. Finally, the density matrix itself can be decomposed into contributions from each order of interaction with the electric

and field and all necessary quantities can then be calculated from this decomposition. The density matrix is,

$$\rho(\boldsymbol{E}) = \rho^{(0)} + \rho^{a}E^{a} + \frac{1}{2!}\rho^{ab}E^{a}E^{b} + \frac{1}{3!}\rho^{abc}E^{a}E^{b}E^{c} + O(E^{4}), \qquad (S6)$$

and inserting into Eq. 5, we obtain the tensor polarizabilities as,

$$P^{a}(\boldsymbol{E}) = -Tr[H^{a}\rho^{0}] - \mathrm{Tr}\left[\mathrm{H^{a}}\rho^{\mathrm{b}}\right]\mathrm{E^{b}} - \frac{1}{2!}\mathrm{Tr}\left[\mathrm{H^{a}}\rho^{\mathrm{bc}}\right]\mathrm{E^{b}E^{c}} - \frac{1}{3!}\mathrm{Tr}\left[\mathrm{H^{a}}\rho^{\mathrm{bcd}}\right]\mathrm{E^{b}E^{c}E^{d}} + \mathrm{O}(\mathbf{E}^{4}), \qquad (S7)$$

with,

$$\begin{gathered} \mu_{a} = -Tr[H^{a}\rho^{0}], \\ \alpha_{ab}(-\omega;\omega) = -Tr[H^{a}\rho^{b}], \\ \beta_{abc}(-2\omega;\omega,\omega) = -Tr[H^{a}\rho^{bc}] \\ \gamma_{abcd}(-3\omega;\omega,\omega,\omega) = -Tr[H^{a}\rho^{bcd}], \end{gathered} \qquad (S8)$$

where the ω frequencies can be either the static frequeny (*i.e.*, 0) or the forward or backward external electric frequencies (*i.e.*, $-\omega, \omega$). Higher-order frequencies that are integer multiples of the external frequency can be calculated using rules from generic perturbation theory to simplify the problem.[14]

The main challenge is obtaining the nth-order density matrix ρ^{n} and is the main inspiration for solving the coupled perturbed Hartree-Fock (CPHF) equations. The explicit details of these equations can be found in ref. [13]. Here, only the main equations in a truncated form will be presented in the language of HF but can be easily adapted for DFT. The Fock matrix equation subject to a time-dependent interaction can be written as,

$$F(\boldsymbol{E})C(\boldsymbol{E}) - i\frac{\partial}{\partial t}SC\epsilon(\boldsymbol{E}) = SC\epsilon(\boldsymbol{E}), \qquad (S9)$$

where C are the time-dependent molecular orbital expansion coefficients, F is the Fock matrix, and S is the overlap matrix of the basis functions. The molecular orbitals obey the following orthogonalization condition: $\frac{\partial}{\partial t}C^{\dagger}SC = 0$. After expanding in orders of electric field interaction, as above, we obtain the CPHF equations for the Fock matrix can be written as,

$$\begin{aligned} F = F^{(0)} &+ \boldsymbol{E}\left[e^{\pm i\omega t}F(\pm\omega) + F(0)\right] \\ &+ \boldsymbol{E}^{2}\left[e^{\pm 2i\omega t}F(\pm\omega,\pm\omega) + e^{\pm i\omega t}F(\pm\omega,0) + 2F(\pm\omega,\mp\omega) + F(0,0)\right] + O(\boldsymbol{E}^{3}), \end{aligned} \qquad (S10)$$

and solving for the frequency-dependent expansion vectors using Eq. 9, one can construct the perturbed density matrix equation as,

$$\begin{aligned} \rho(\boldsymbol{E}) &= C(\boldsymbol{E})nC^{\dagger}(\boldsymbol{E}) \\ &= \rho^{(0)} + \boldsymbol{E}\left[e^{\pm i\omega t}\rho(\pm\omega) + \rho(0)\right] \\ +\boldsymbol{E}^2\left[e^{\pm 2i\omega t}\rho(\pm\omega,\pm\omega) + e^{\pm i\omega t}\rho(\pm\omega,0) + 2\rho(\pm\omega,\mp\omega) + \rho(0)\right] &+ O(\boldsymbol{E}^3). \end{aligned} \qquad (S11)$$

Once the perturbed density matrix is calculated, the tensor polarizabilities can be obtained through various orders of differentiation of the density matrix with respect to the electric field. In principle, these four tensors allow one to calculate a variety of non-linear spectra. In the present work, we limit ourselves to the calculation of Raman spectroscopy, which will only make use of the polarizability α_{ab}. To make a simple connection to Raman spectroscopy, the state-to-state polarizability can be calculated in the language of normal modes $\{Q_n\}$ as,

$$[\alpha_{ab}]_{nm} = (\alpha_{ab})_0\langle m|n\rangle + \frac{\partial\alpha_{ab}}{\partial Q_l}\langle m|Q_l|n\rangle + O(Q^2), \qquad (S12)$$

Where $|\,n\,\rangle$ and $|\,m\,\rangle$ are the initial and final states, respectively. This expressions indicates that the Raman intensity will only be non-zero for a n-to-m transition if there exists a change in the polarizability with respect to nuclear configuration as well as having an appreciable dipole moment between n and m states. This quantity can often be obtained from analytical gradient expressions with respect to nuclear coordinates implemented in many software packages for HF and DFT levels of theory.

Normal Mode Character

The normal modes of each model (pristine and defected) were extracted with the vectorial expansion coefficients for each atom written as,

$$\boldsymbol{V}_k^{\alpha} = < D_{k,x}^{\alpha}, D_{k,y}^{\alpha}, D_{k,z}^{\alpha} >, \qquad (S13)$$

where α is a nucleus and k is the normal mode index. The norm of each atom contribution is then computed as,

$$V_k^{\alpha} = \sum_{d=x,y,z}\left(D_{k,d}^{\alpha}\right)^2. \qquad (S14)$$

A discrete probability distribution $P_k(X_b) = C_k(X_b)^2$ can be constructed by binning the contributions from all atoms within a spatial histogram $C_k(\mathrm{X_b})$ at bin $\mathrm{X_b}$ of width dX = 0.45 Å (100 bins spanning the length of the SWCNT main axis) for each mode k defined as,

$$C_k(X_b) = \sum_{\alpha}^{N_{Atoms}} V_k^{\alpha}, \qquad (S15)$$

for all α in bin X_b. The normalization of the probability distribution $P_k(X_b)$ for each mode k is defined as,

$$1 = \sum_{b=1}^{N_{Bins}} \mathrm{P_k}(X_b) = \sum_{b=1}^{N_{Bins}} C_k(X_b)^2. \qquad (S16)$$

This probability distribution will be referred to as a position-resolved normal mode probability distribution and will be able to give insight into the spatial localization of each normal mode.

Fig. S5 presents the amplitude distributions for each mode in the (a) pristine and (b) defected SWCNT models, showcasing different localization features of the modes.

Fig. S6 presents the probability amplitude of the normal modes as a function of position and normal mode index for the (a) pristine and (b) defected SWCNT models, which is just an extension of Fig. S5 now as a 2D map instead of overlayed functions.

Fig. 5 in the main text is an extension of this plot, but now as an amplitude density map where the normal modes probability amplitudes have been convolved with a finite-width Gaussian function defined as,

$$C(E, X_b) = \sum_{k=0}^{N_{Modes}} C_k(X_b) e^{-\frac{(E-E_k)^2}{2\sigma^2}}, \qquad (S17)$$

where $\sigma = 5$ cm^{-1}. This allows to visualize the spatial distribution of the modes as a function of the normal mode frequency. Fig. S7 shows the same data as a 3D contour plot for visual comparison to the 2D density map in Fig. 5 in the main text for the defected SWCNT.

To gain a better understanding of which types of mode characters are contributing to the intensity of the CPHF spectrum, we further decompose the position-resolved probability distribution into three classes of modes: (I) Edge-localized, (II) Defect-(center-)localized, and (III) all others.

If the normal mode is localized to within 5 Å of the edge by more than 35 % (sum of the probabilities of both edges), the intensity of this mode for each value of CPHF energy is shown as the percent of the maximum intensity in the system in Fig. 5 for the (a) pristine and (b) defected SWCNT models. If the normal mode is localized to the center 10 Å (5 Å on either side of the center of the SWCNT) by more than 30 %, the intensity of this mode for each value of CPHF energy is shown as the percent of the maximum intensity in the system in Fig. 5 for the (c) pristine and (d) defected SWCNT models.

One can define a localization parameter, similar to that of the inverse participation ratio commonly used in the description of quantum delocalization.[1–4] We define the L_d parameter for the kth normal mode as,

$$(L_d)_k = \left[\sum_{b=0}^{N_{Bins}} P_k(X_b)\right]^{-1}, \qquad (S18)$$

which can be converted to length by $(L_d)_k \rightarrow (L_d)_k * dX$ or to a percent of the total SWCNT axis as $(L_d)_k \rightarrow (L_d)_k * \frac{dX}{L_x}$, where L_x is the length of the SWCNT axis. Here, the larger the percent $(L_d)_k$ (%) the more delocalized the mode along the SWCNT axis; whereas, a small percent indicates that the mode is localized to only that percent of the SWCNT. This data is shown in Fig. S9.

References

(1) Tretiak, S.; Mukamel, S. Density Matrix Analysis and Simulation of Electronic Excitations in Conjugated and Aggregated Molecules. *Chem. Rev.* **2002**, *102* (9), 3171–3212.
(2) Kilina, S.; Batista, E. R.; Yang, P.; Tretiak, S.; Saxena, A.; Martin, R. L.; Smith, D. L. Electronic Structure of Self-Assembled Amorphous Polyfluorenes. *ACS Nano* **2008**, *2* (7), 1381–1388.
(3) Murphy, N. C.; Wortis, R.; Atkinson, W. A. Generalized Inverse Participation Ratio as a Possible Measure of Localization for Interacting Systems. *Phys. Rev. B* **2011**, *83* (18).
(4) Wegner, F. Inverse Participation Ratio in 2 + e Dimensions. *Z. Physik B Cond. Mat.* **1980**, *36* (3), 209–214.
(5) McWeeny, R. Some Recent Advances in Density Matrix Theory. *Rev. Mod. Phys.* **1960**, *32* (2), 335–369.
(6) McWeeny, R. Perturbation Theory for the Fock-Dirac Density Matrix. *Phys. Rev.* **1962**, *126* (3), 1028–1034.
(7) Stevens, R. M.; Pitzer, R. M.; Lipscomb, W. N. Perturbed Hartree—Fock Calculations. I. Magnetic Susceptibility and Shielding in the LiH Molecule. *J. Chem. Phys.* **1963**, *38* (2), 550–560.
(8) Gerratt, J.; Mills, I. M. Force Constants and Dipole-Moment Derivatives of Molecules from Perturbed Hartree–Fock Calculations. I. *J. Chem. Phys.* **1968**, *49* (4), 1719–1729.
(9) Dodds, J. L.; McWeeny, R.; Raynes, W. T.; Riley, J. P. SCF Theory for Multiple Perturbations. *Mol. Phys.* **1977**, *33* (3), 611–617.
(10) Dodds, J. L.; McWeeny, R.; Sadlej, A. J. Self-Consistent Perturbation Theory: Generalization for Perturbation-Dependent Non-Orthogonal Basis Set† This Research Was Partly Supported by the Institute of Low Temperatures and Structure Research of the Polish Academy of Sciences. *Mol. Phys.* **1977**, *34* (6), 1779–1791.
(11) Dykstra, C. E.; Jasien, P. G. Derivative Hartree—Fock Theory to All Orders. *Chem. Phys. Lett.* **1984**, *109* (4), 388–393.
(12) Karna, S. P.; Prasad, P. N.; Dupuis, M. Nonlinear Optical Properties of *p* -nitroaniline: An *Ab Initio* Time-dependent Coupled Perturbed Hartree–Fock Study. *J. of Chem. Phys.* **1991**, *94* (2), 1171–1181.
(13) Sekino, H.; Bartlett, R. J. Frequency Dependent Nonlinear Optical Properties of Molecules. *J. of Chem. Phys.* **1986**, *85* (2), 976–989.

(14) Karna, S. P. Spin-unrestricted Time-dependent Hartree–Fock Theory of Frequency-dependent Linear and Nonlinear Optical Properties. *J. of Chem. Phys.* **1996**, *104* (17), 6590–6605.

	**Defected SWCNT Model**	
**Mode Label**	**FREQ(cm^-1)**	**Ld (%)**
1	33.4	78.6
2	33.7	79.2
3	52.7	48.5
4	52.8	49.4
5	56.0	77.0
6	59.2	83.3
7	60.1	85.0
8	63.5	82.6
9	63.8	82.9
10	71.5	76.9
11	72.5	77.8
12	75.5	87.2
13	76.4	82.0
14	84.6	64.9
15	87.2	66.7
16	91.8	68.2
17	92.2	62.1
18	95.1	38.2
19	102.8	46.1
20	107.9	57.9
21	111.4	74.6
22	111.9	79.1
23	117.3	72.7
24	121.8	81.1
25	127.7	71.3
26	136.5	78.5
27	141.5	73.8
28	152.4	85.1
29	155.4	79.9
30	158.9	87.3
31	159.8	92.9
32	162.2	80.5
33	162.6	87.8
34	164.6	92.6
35	164.7	83.6
36	166.9	82.8
37	169.4	83.9
38	169.7	87.1
39	173.7	91.7

40	176.9	87.5
41	180.6	81.0
42	185.5	90.4
43	187.2	87.7
44	190.8	85.5
45	192.5	81.9
46	192.8	89.0
47	197.4	83.5
48	200.9	86.2
49	201.5	91.8
50	206.0	79.3
51	208.6	88.8
52	210.1	74.8
53	214.5	76.1
54	216.1	81.5
55	218.1	73.2
56	220.5	81.6
57	225.5	72.7
58	228.4	78.9
59	228.4	79.8
60	230.6	84.4
61	232.5	58.2
62	232.7	47.2
63	238.6	53.8
64	239.8	30.9
65	243.0	60.5
66	245.3	42.6
67	246.2	78.9
68	248.3	76.4
69	248.6	76.6
70	252.1	69.1
71	253.8	78.4
72	254.1	88.7
73	254.7	88.3
74	257.6	84.0
75	259.7	72.3
76	263.7	57.2
77	266.0	63.6
78	267.2	74.2
79	269.1	62.7
80	272.5	85.3
81	275.9	85.1
82	276.9	82.4
83	278.2	76.8
84	279.9	86.2
85	280.2	86.7

86	282.1	86.9
87	285.2	82.8
88	287.9	84.2
89	288.8	85.6
90	290.4	82.7
91	294.0	85.3
92	294.0	91.6
93	295.0	79.1
94	297.9	83.6
95	297.9	88.9
96	299.7	92.9
97	303.8	85.8
98	304.2	85.3
99	305.9	83.9
100	307.2	89.3
101	307.9	81.9
102	308.2	85.0
103	309.8	89.6
104	310.1	78.9
105	311.7	78.7
106	312.3	85.6
107	314.5	83.4
108	314.7	86.0
109	318.5	89.9
110	322.0	87.9
111	323.1	85.3
112	324.1	85.2
113	325.2	82.5
114	331.7	88.8
115	332.1	86.0
116	333.4	85.8
117	334.8	89.2
118	335.3	84.5
119	335.7	85.0
120	339.1	83.4
121	339.5	86.8
122	340.5	70.3
123	342.5	83.5
124	343.2	76.4
125	344.1	90.8
126	347.1	85.4
127	347.3	89.8
128	354.5	88.9
129	355.9	85.4
130	357.7	84.1
131	358.2	88.6

132	359.2	84.1
133	361.3	82.5
134	363.7	89.1
135	365.6	84.7
136	366.9	87.9
137	369.4	88.2
138	370.2	81.4
139	372.3	83.2
140	375.3	83.3
141	379.6	83.2
142	380.3	88.6
143	380.8	83.6
144	381.3	84.0
145	384.2	81.9
146	386.8	85.9
147	387.8	90.4
148	389.2	92.3
149	389.6	88.2
150	390.5	91.8
151	391.9	88.3
152	392.4	88.4
153	395.1	89.4
154	396.6	89.2
155	397.0	88.8
156	399.0	91.6
157	400.3	90.9
158	401.7	85.8
159	402.1	86.5
160	402.6	90.7
161	405.0	91.0
162	406.1	89.0
163	406.5	86.1
164	407.7	83.9
165	410.9	89.8
166	413.3	86.4
167	413.9	91.1
168	415.1	87.8
169	416.3	89.8
170	416.4	88.3
171	417.9	88.2
172	419.0	89.2
173	422.0	89.4
174	422.1	86.1
175	423.0	90.8
176	424.9	89.2
177	425.2	87.5

178	427.1	87.2
179	427.7	91.6
180	428.1	90.1
181	430.6	84.1
182	431.1	89.3
183	431.9	92.4
184	432.9	90.2
185	433.0	92.2
186	433.7	92.2
187	435.4	88.3
188	435.5	91.5
189	436.3	88.2
190	439.0	86.3
191	439.7	90.7
192	441.2	85.2
193	441.5	89.3
194	442.9	85.8
195	444.0	81.7
196	446.2	89.7
197	447.8	80.7
198	452.0	87.8
199	453.1	89.0
200	453.4	83.2
201	454.5	89.6
202	456.2	91.3
203	457.5	86.4
204	457.8	85.1
205	459.0	90.7
206	459.1	88.5
207	460.4	92.9
208	461.3	88.3
209	462.1	91.6
210	463.6	87.2
211	464.1	89.0
212	464.7	89.3
213	465.5	94.0
214	466.7	85.6
215	466.9	85.8
216	467.7	79.7
217	468.5	91.7
218	469.0	88.3
219	469.1	88.5
220	473.8	86.7
221	475.1	86.0
222	476.4	86.1
223	476.8	87.2

224	477.6	72.6
225	478.1	70.9
226	478.3	63.5
227	480.5	81.4
228	480.6	83.2
229	482.4	87.8
230	482.6	91.3
231	486.3	88.1
232	487.0	84.0
233	490.7	85.1
234	492.1	84.6
235	492.8	86.0
236	494.2	92.2
237	499.2	92.1
238	499.9	93.1
239	500.7	91.3
240	502.7	83.4
241	503.7	91.4
242	504.3	89.8
243	506.7	91.1
244	512.5	89.8
245	512.8	85.9
246	516.1	87.6
247	517.4	86.1
248	518.2	83.0
249	521.8	91.9
250	522.7	89.6
251	525.7	91.5
252	525.9	89.7
253	529.4	92.0
254	531.6	87.8
255	534.1	85.8
256	535.9	83.8
257	536.6	82.5
258	539.4	86.3
259	542.0	77.6
260	544.5	89.7
261	545.1	88.2
262	547.4	86.4
263	548.0	88.9
264	548.8	90.2
265	550.7	83.0
266	552.4	81.4
267	554.7	90.2
268	559.0	86.9
269	559.9	90.2

270	560.7	89.7
271	562.9	74.6
272	566.5	57.3
273	566.7	86.5
274	568.2	91.9
275	569.5	88.5
276	571.6	80.5
277	573.1	74.8
278	574.4	89.3
279	576.7	81.7
280	577.4	90.6
281	579.0	89.3
282	579.3	82.6
283	581.1	92.1
284	581.8	87.6
285	583.4	87.3
286	585.5	86.1
287	585.5	90.9
288	588.0	84.0
289	588.0	90.6
290	589.3	78.5
291	589.7	75.5
292	590.2	83.5
293	590.5	86.6
294	591.4	85.9
295	592.1	90.6
296	593.0	92.1
297	594.8	92.9
298	597.1	91.9
299	598.7	87.1
300	599.4	90.1
301	601.0	92.1
302	602.0	89.7
303	604.1	84.5
304	604.5	90.8
305	605.2	85.8
306	605.8	91.7
307	608.1	77.4
308	608.5	83.0
309	609.8	84.2
310	610.2	90.3
311	612.3	90.5
312	612.5	89.6
313	613.4	83.4
314	614.2	91.6
315	614.3	91.7

316	614.7	87.9
317	614.9	91.3
318	616.4	87.1
319	618.9	87.7
320	619.0	93.2
321	620.8	91.9
322	621.2	92.1
323	621.5	85.1
324	621.6	91.5
325	624.0	84.0
326	624.1	86.9
327	625.0	89.7
328	626.7	88.4
329	628.7	89.6
330	629.5	80.3
331	629.7	89.2
332	630.9	90.6
333	631.9	88.1
334	632.7	90.2
335	633.5	91.8
336	634.9	89.5
337	635.0	88.6
338	635.8	89.4
339	636.3	92.2
340	636.8	89.3
341	637.7	89.6
342	639.2	92.8
343	639.8	92.9
344	640.0	87.7
345	641.0	89.9
346	641.4	93.2
347	642.6	91.7
348	642.7	88.8
349	643.9	87.2
350	644.4	90.2
351	644.9	89.7
352	645.8	91.1
353	647.4	86.8
354	648.8	89.3
355	649.4	82.9
356	649.9	91.5
357	650.3	93.5
358	651.6	87.7
359	653.1	92.5
360	653.2	88.6
361	654.3	87.9

362	655.0	91.7
363	656.1	92.8
364	656.8	92.8
365	657.3	88.6
366	658.0	89.9
367	658.7	92.1
368	659.4	94.1
369	660.5	92.7
370	661.0	87.5
371	662.1	86.8
372	663.7	93.0
373	664.0	92.3
374	665.5	90.7
375	666.6	89.6
376	667.3	89.8
377	667.3	75.9
378	667.6	86.5
379	668.8	92.0
380	669.7	89.5
381	670.0	91.8
382	670.7	78.3
383	671.0	77.4
384	671.5	88.1
385	673.1	82.5
386	674.1	90.2
387	675.1	89.3
388	676.6	86.9
389	679.0	81.1
390	679.5	89.9
391	681.0	86.9
392	681.7	89.0
393	683.0	90.6
394	684.5	80.8
395	685.7	86.3
396	685.7	85.4
397	687.3	89.0
398	688.8	88.6
399	690.0	90.3
400	690.5	91.5
401	690.5	90.6
402	691.8	93.3
403	692.7	89.5
404	693.5	89.3
405	693.9	85.5
406	695.4	88.8
407	696.3	92.6

408	697.6	91.8
409	697.9	89.3
410	700.1	91.7
411	700.3	89.7
412	700.7	86.0
413	700.8	86.5
414	702.7	89.9
415	704.6	89.0
416	705.8	83.8
417	706.2	91.2
418	708.2	91.1
419	708.5	89.3
420	709.3	89.6
421	709.6	88.7
422	710.1	86.8
423	711.0	92.1
424	712.0	86.7
425	714.4	86.8
426	714.5	89.1
427	716.4	91.9
428	717.8	89.0
429	718.1	88.5
430	718.7	87.9
431	719.2	84.7
432	722.2	89.3
433	722.2	93.0
434	724.0	81.8
435	724.4	87.5
436	725.9	84.4
437	727.5	88.9
438	727.6	90.7
439	729.0	87.5
440	729.2	91.0
441	731.1	83.6
442	731.8	82.8
443	732.5	85.8
444	734.0	81.3
445	734.5	91.0
446	736.1	77.1
447	736.3	85.7
448	737.0	87.4
449	739.1	89.5
450	740.0	90.8
451	740.2	92.0
452	742.4	65.2
453	742.8	83.3

454	744.5	67.8
455	744.7	92.2
456	746.7	78.9
457	746.9	78.9
458	747.2	44.1
459	748.2	86.4
460	750.1	85.4
461	750.3	78.5
462	750.5	80.6
463	753.4	79.5
464	753.5	87.6
465	755.3	89.9
466	757.9	84.0
467	758.3	72.3
468	758.4	87.9
469	758.7	82.7
470	760.3	89.5
471	760.8	83.5
472	761.4	83.3
473	764.6	67.6
474	764.9	87.4
475	765.9	68.2
476	768.3	71.6
477	771.5	57.1
478	771.5	77.4
479	772.2	81.2
480	774.3	74.2
481	774.5	87.1
482	775.3	82.0
483	775.6	82.6
484	778.1	85.7
485	778.6	79.6
486	779.7	89.6
487	780.2	90.2
488	780.9	65.5
489	781.5	61.2
490	782.1	77.6
491	784.1	85.9
492	785.5	87.4
493	786.3	84.1
494	787.4	75.0
495	788.2	90.7
496	788.9	49.1
497	789.5	84.6
498	791.0	91.8
499	791.6	85.5

500	793.1	68.3
501	794.0	61.4
502	794.9	85.9
503	795.9	52.0
504	796.2	53.4
505	797.1	68.6
506	798.0	78.8
507	798.1	87.1
508	798.5	78.0
509	799.0	73.0
510	800.7	88.3
511	801.2	66.0
512	802.4	82.1
513	803.1	78.5
514	803.8	74.4
515	804.4	69.1
516	805.5	65.3
517	805.7	86.7
518	806.3	67.2
519	807.1	79.0
520	807.4	77.0
521	808.4	90.1
522	809.2	67.7
523	809.9	63.3
524	810.7	81.1
525	810.8	82.3
526	811.4	81.8
527	812.5	83.3
528	813.0	90.0
529	813.6	82.1
530	814.5	82.7
531	815.0	73.8
532	816.9	83.6
533	819.3	86.9
534	822.7	84.2
535	823.4	86.7
536	823.5	88.0
537	824.9	90.3
538	825.4	88.0
539	826.2	90.1
540	827.6	76.8
541	828.5	88.4
542	830.8	81.7
543	831.0	89.8
544	831.2	87.6
545	831.7	83.8

546	832.9	85.5
547	834.6	88.9
548	835.1	92.8
549	835.6	86.0
550	836.3	89.7
551	836.7	89.9
552	838.5	84.4
553	839.7	81.3
554	840.1	89.7
555	840.1	90.2
556	841.0	91.8
557	841.3	87.7
558	843.6	89.9
559	844.2	89.0
560	844.4	89.7
561	844.6	90.1
562	845.6	83.9
563	846.0	84.7
564	846.8	87.5
565	847.1	82.3
566	847.6	81.5
567	848.0	86.6
568	849.3	83.6
569	851.2	80.4
570	852.5	83.9
571	853.6	89.7
572	853.9	88.3
573	854.4	91.3
574	855.2	87.1
575	856.3	86.3
576	856.9	79.5
577	858.4	92.1
578	858.7	78.8
579	860.0	79.1
580	860.4	87.6
581	860.7	79.9
582	861.1	76.0
583	862.2	78.2
584	862.7	71.1
585	863.4	75.7
586	863.7	91.4
587	863.8	64.4
588	864.0	77.3
589	864.1	78.0
590	865.7	87.1
591	866.3	74.1

592	866.3	72.9
593	867.6	64.2
594	867.6	68.0
595	868.1	87.0
596	871.2	86.7
597	871.3	88.7
598	872.9	89.9
599	877.3	86.3
600	882.1	86.2
601	884.2	81.3
602	891.4	86.9
603	891.6	85.3
604	895.9	67.2
605	897.1	80.3
606	897.3	89.2
607	899.7	42.8
608	900.4	53.0
609	902.2	54.2
610	902.4	58.7
611	905.7	77.7
612	910.6	87.2
613	914.5	74.0
614	919.1	22.3
615	919.5	17.0
616	922.1	75.6
617	925.7	78.5
618	926.4	63.7
619	927.5	88.5
620	930.5	24.1
621	930.6	13.5
622	933.0	74.1
623	934.2	67.8
624	937.6	11.6
625	937.9	29.7
626	939.6	40.8
627	939.8	84.7
628	945.4	77.0
629	947.4	35.4
630	949.4	27.6
631	950.4	70.5
632	952.8	51.9
633	952.9	43.7
634	955.1	8.2
635	955.8	16.6
636	958.9	39.6
637	960.0	8.4

638	960.3	9.0
639	960.9	18.3
640	961.7	44.0
641	962.7	13.1
642	962.8	14.5
643	964.8	79.0
644	965.8	35.9
645	966.3	16.7
646	967.6	41.3
647	969.5	67.6
648	970.5	81.7
649	974.6	84.8
650	976.4	84.6
651	978.4	87.1
652	980.6	91.7
653	988.2	82.5
654	992.4	86.3
655	992.6	86.8
656	994.7	79.2
657	997.3	80.0
658	998.5	90.2
659	1005.7	90.0
660	1006.5	90.4
661	1007.1	87.3
662	1017.3	66.2
663	1018.8	54.4
664	1020.6	50.5
665	1025.5	68.4
666	1026.1	79.8
667	1027.4	71.1
668	1034.6	52.8
669	1036.4	57.5
670	1037.4	72.8
671	1041.8	76.0
672	1042.1	61.1
673	1050.7	82.0
674	1051.2	65.2
675	1051.6	86.6
676	1053.3	75.7
677	1056.5	80.2
678	1059.8	88.6
679	1068.4	81.0
680	1069.3	81.9
681	1074.9	86.4
682	1079.3	90.0
683	1082.7	72.0

684	1085.5	79.6
685	1091.0	78.8
686	1094.4	86.3
687	1098.2	86.3
688	1105.7	84.8
689	1109.3	86.3
690	1112.2	90.9
691	1113.1	85.1
692	1115.6	83.7
693	1118.3	76.5
694	1119.3	78.8
695	1126.6	88.0
696	1135.1	91.0
697	1141.4	77.0
698	1142.5	79.3
699	1145.0	81.7
700	1145.9	78.9
701	1147.0	89.1
702	1153.1	79.2
703	1157.9	88.3
704	1158.2	77.0
705	1160.3	74.6
706	1165.7	74.0
707	1167.0	88.1
708	1169.2	87.1
709	1172.7	70.4
710	1173.3	77.0
711	1178.1	80.5
712	1179.5	80.4
713	1183.1	83.4
714	1185.1	71.8
715	1188.3	86.7
716	1193.2	74.3
717	1194.1	72.2
718	1196.3	80.0
719	1196.5	75.1
720	1199.8	84.5
721	1201.8	74.9
722	1209.0	69.9
723	1210.5	69.3
724	1213.5	71.7
725	1217.4	68.5
726	1217.9	67.9
727	1219.9	59.3
728	1222.4	83.5
729	1223.3	79.0

730	1224.1	74.7
731	1225.3	82.8
732	1230.8	74.8
733	1233.3	83.8
734	1234.7	75.7
735	1236.8	77.4
736	1237.9	72.1
737	1239.7	88.1
738	1241.3	55.5
739	1244.7	58.3
740	1246.2	73.5
741	1248.8	59.4
742	1252.3	73.3
743	1252.7	77.5
744	1256.4	73.5
745	1256.5	80.2
746	1260.9	74.7
747	1261.6	63.3
748	1265.0	88.4
749	1265.2	85.9
750	1266.5	79.5
751	1268.0	75.1
752	1269.2	82.0
753	1270.1	71.8
754	1271.2	70.7
755	1273.7	88.2
756	1275.5	85.5
757	1275.7	86.3
758	1279.6	57.9
759	1280.3	79.7
760	1280.9	83.3
761	1282.6	81.4
762	1285.7	63.3
763	1286.8	74.3
764	1288.7	78.4
765	1290.3	85.3
766	1291.0	81.4
767	1292.5	67.5
768	1293.8	78.1
769	1297.0	73.4
770	1300.2	54.6
771	1300.6	78.3
772	1304.1	77.1
773	1307.2	66.6
774	1309.4	71.2
775	1311.8	76.0

776	1312.0	75.8
777	1313.7	89.0
778	1315.9	84.0
779	1316.2	74.3
780	1319.4	79.1
781	1320.4	80.3
782	1321.3	72.2
783	1322.5	78.7
784	1325.3	82.1
785	1326.3	93.2
786	1326.4	75.1
787	1328.1	84.5
788	1330.2	79.7
789	1331.4	66.8
790	1332.1	85.1
791	1334.5	65.0
792	1335.9	71.3
793	1336.1	68.9
794	1336.7	73.4
795	1340.2	88.4
796	1340.6	72.3
797	1341.8	76.9
798	1344.1	85.7
799	1344.2	81.3
800	1345.2	82.1
801	1345.7	87.9
802	1346.6	71.1
803	1349.2	80.7
804	1349.5	76.9
805	1353.3	89.2
806	1355.2	91.4
807	1357.1	76.7
808	1357.8	90.3
809	1359.6	90.1
810	1362.5	80.4
811	1362.6	89.5
812	1364.0	87.9
813	1364.4	91.7
814	1366.4	88.1
815	1367.7	86.5
816	1368.4	83.6
817	1370.6	75.0
818	1370.8	91.9
819	1372.1	82.4
820	1373.5	90.8
821	1374.9	86.0

822	1376.1	66.5
823	1378.2	82.3
824	1380.9	89.0
825	1382.6	74.8
826	1383.2	86.9
827	1386.7	91.5
828	1388.4	90.1
829	1388.8	83.7
830	1389.8	88.8
831	1390.4	81.4
832	1390.7	83.6
833	1391.9	86.6
834	1394.8	92.4
835	1395.4	82.9
836	1396.4	87.6
837	1397.1	90.3
838	1398.0	88.7
839	1399.3	88.1
840	1399.7	83.5
841	1401.3	84.2
842	1403.6	83.7
843	1403.7	82.8
844	1404.5	89.2
845	1405.1	91.1
846	1405.1	87.8
847	1405.9	89.3
848	1407.4	87.2
849	1408.6	88.2
850	1408.6	84.0
851	1410.8	79.0
852	1411.4	88.7
853	1411.9	89.8
854	1413.1	88.8
855	1413.5	90.8
856	1414.9	88.9
857	1414.9	85.6
858	1416.2	90.2
859	1416.9	85.3
860	1418.3	82.9
861	1419.0	87.7
862	1420.0	88.4
863	1421.1	83.4
864	1421.6	87.8
865	1421.9	88.8
866	1422.3	85.7
867	1423.4	87.7

868	1423.8	87.8
869	1424.1	90.3
870	1424.8	84.6
871	1425.8	89.9
872	1426.0	88.6
873	1426.2	86.3
874	1427.3	91.0
875	1427.4	87.5
876	1428.0	89.9
877	1428.6	89.9
878	1428.9	76.3
879	1429.1	88.0
880	1429.2	88.3
881	1430.3	87.5
882	1430.4	88.1
883	1431.6	87.8
884	1431.9	87.7
885	1432.7	81.5
886	1433.8	86.6
887	1434.1	85.4
888	1434.8	88.8
889	1434.9	87.9
890	1435.1	88.1
891	1435.6	87.1
892	1437.2	86.9
893	1437.7	83.2
894	1438.2	80.9
895	1438.5	81.1
896	1438.7	84.4
897	1439.4	89.1
898	1439.8	85.5
899	1440.5	89.4
900	1441.9	87.6
901	1442.3	84.2
902	1442.4	88.0
903	1443.4	88.4
904	1443.5	91.5
905	1443.9	85.9
906	1444.5	86.9
907	1444.8	84.8
908	1445.1	74.5
909	1445.3	84.1
910	1445.7	88.0
911	1446.6	84.2
912	1446.6	87.7
913	1447.4	88.8

914	1448.2	77.5
915	1448.2	89.0
916	1448.9	87.5
917	1449.3	83.6
918	1449.9	80.4
919	1451.6	90.0
920	1451.8	89.2
921	1452.8	84.2
922	1453.3	91.3
923	1453.7	90.6
924	1453.7	91.0
925	1454.8	86.3
926	1454.9	88.7
927	1456.2	89.4
928	1456.4	89.9
929	1458.3	86.9
930	1458.7	86.6
931	1459.0	87.2
932	1459.2	91.4
933	1459.6	90.6
934	1461.5	87.7
935	1462.6	78.4
936	1462.9	91.0
937	1463.7	84.5
938	1465.0	85.3
939	1466.0	82.5
940	1466.0	80.5
941	1466.5	82.5
942	1468.6	92.2
943	1470.0	89.0
944	1470.5	82.5
945	1471.8	87.5
946	1473.0	89.7
947	1473.5	88.3
948	1474.0	80.3
949	1475.0	81.6
950	1475.9	86.3
951	1478.0	92.5
952	1478.5	71.4
953	1478.8	78.0
954	1480.3	84.1
955	1480.9	92.2
956	1481.1	89.0
957	1483.5	87.7
958	1484.1	72.3
959	1484.8	89.5

960	1484.9	88.1
961	1486.9	90.0
962	1488.2	90.2
963	1488.4	91.2
964	1488.8	90.4
965	1489.1	90.4
966	1491.8	91.0
967	1492.5	89.8
968	1493.9	91.1
969	1495.1	88.8
970	1496.4	81.3
971	1497.4	88.7
972	1497.5	87.0
973	1498.6	87.1
974	1500.1	90.3
975	1500.3	89.7
976	1501.4	91.1
977	1502.3	91.1
978	1503.7	84.1
979	1505.1	91.5
980	1505.8	91.5
981	1507.3	91.5
982	1508.5	89.9
983	1511.6	86.3
984	1511.9	91.7
985	1513.3	83.6
986	1514.6	88.9
987	1515.0	85.6
988	1517.6	90.5
989	1517.7	88.0
990	1519.0	87.7
991	1519.2	91.2
992	1521.4	90.2
993	1522.3	88.4
994	1522.5	86.0
995	1522.7	91.6
996	1525.5	92.8
997	1526.7	89.6
998	1528.1	86.9
999	1529.2	74.2
1000	1530.2	82.5
1001	1532.1	88.7
1002	1532.8	72.6
1003	1533.4	91.0
1004	1534.9	78.2
1005	1536.5	73.9

1006	1537.3	81.7
1007	1537.7	90.5
1008	1538.0	79.4
1009	1538.2	85.9
1010	1539.2	82.7
1011	1540.7	73.1
1012	1543.0	74.6
1013	1543.5	64.8
1014	1543.8	86.5
1015	1543.9	78.8
1016	1544.2	88.2
1017	1544.8	75.7
1018	1545.6	84.3
1019	1547.7	82.2
1020	1548.0	75.4
1021	1549.2	64.2
1022	1550.0	49.1
1023	1550.3	83.5
1024	1550.5	41.1
1025	1550.5	60.2
1026	1551.6	67.5
1027	1552.6	73.4
1028	1553.0	72.3
1029	1553.7	59.8
1030	1553.8	79.0
1031	1554.6	57.7
1032	1555.4	80.5
1033	1556.3	39.7
1034	1556.7	58.3
1035	1556.9	71.8
1036	1559.4	65.4
1037	1560.0	41.1
1038	1560.4	68.9
1039	1561.7	61.8
1040	1562.7	73.1
1041	1565.6	68.4
1042	1567.5	81.3
1043	1568.1	53.9
1044	1569.4	68.6
1045	1570.2	87.3
1046	1574.3	88.4
1047	1574.6	72.5
1048	1575.4	90.4
1049	1575.7	86.2
1050	1579.1	92.8
1051	1580.7	74.8

1052	1583.0	89.3
1053	1583.3	76.0
1054	1586.3	90.8
1055	1588.2	87.9
1056	1589.6	85.8
1057	1590.5	88.0
1058	1591.1	90.7
1059	1591.7	77.5
1060	1595.0	81.4
1061	1595.6	85.6
1062	1598.0	85.0
1063	1598.2	87.8
1064	1599.1	91.6
1065	1601.6	88.6
1066	1603.7	86.9
1067	1604.8	87.4
1068	1607.6	80.7
1069	1609.2	90.8
1070	1609.6	86.2
1071	1610.7	89.8
1072	1611.9	87.8
1073	1612.4	90.0
1074	1613.6	84.8
1075	1614.1	87.8
1076	1615.0	90.7
1077	1617.0	87.9
1078	1620.3	89.8
1079	1622.0	86.1
1080	1622.5	88.5
1081	1624.4	88.8
1082	1624.6	91.4
1083	1625.8	86.1
1084	1628.4	86.8
1085	1629.0	88.2
1086	1629.8	81.9
1087	1630.5	89.0
1088	1631.4	89.5
1089	1631.7	71.6
1090	1633.2	82.5
1091	1637.3	82.3
1092	1639.4	85.4
1093	1642.0	88.3
1094	1646.9	89.6
1095	1647.8	90.3
1096	1649.5	87.6
1097	1651.4	50.6

1098	1651.6	47.7
1099	1652.6	84.0
1100	1654.9	88.4
1101	1657.1	89.7
1102	1657.6	89.7
1103	1658.1	87.6
1104	1658.8	91.2
1105	1660.5	85.4
1106	1661.7	82.7
1107	1662.2	89.0
1108	1662.4	88.0
1109	1662.6	86.7
1110	1663.3	84.0
1111	1663.4	90.1
1112	1663.6	85.3
1113	1664.3	87.0
1114	1664.6	82.0
1115	1666.2	83.5
1116	1666.7	88.6
1117	1667.0	86.0
1118	1667.1	89.1
1119	1668.9	86.0
1120	1669.2	84.8
1121	1669.5	81.7
1122	1670.2	60.2
1123	1670.7	81.4
1124	1672.0	77.0
1125	1673.3	87.5
1126	1675.1	79.7
1127	1676.0	88.1
1128	1676.9	85.9
1129	1677.7	88.8
1130	1679.8	73.2
1131	1680.4	85.2
1132	1682.0	88.4
1133	1682.8	78.1
1134	1683.2	78.4
1135	1685.0	75.0
1136	1685.4	83.4
1137	1687.5	85.4
1138	1687.7	79.5
1139	1689.2	89.0
1140	1690.3	84.5
1141	1690.4	85.0
1142	1690.8	70.8
1143	1692.6	51.1

1144	1692.8	71.8
1145	1694.8	72.7
1146	1695.4	75.1
1147	1698.1	27.0
1148	1698.1	26.3
1149	1709.9	36.3
1150	1709.9	37.1
1151	1758.4	6.4
1152	1758.4	4.5
1153	1800.4	3.7
1154	1800.4	3.7
1155	1810.8	3.6
1156	1810.9	3.6
1157	3394.5	4.6
1158	3394.5	3.6
1159	3394.7	3.8
1160	3394.7	4.1
1161	3395.1	3.0
1162	3395.1	3.1
1163	3446.3	2.8
1164	3446.4	2.3
1165	3446.4	3.3
1166	3446.5	2.9
1167	3450.7	1.8
1168	3450.7	1.8
1169	3451.5	4.2
1170	3451.5	4.2
1171	3452.1	4.6
1172	3452.1	4.6
1173	3452.4	1.3
1174	3452.4	1.3
1175	3459.4	2.3
1176	3459.4	2.3
1177	3548.1	1.2
1178	3548.1	1.2
1179	3548.7	1.2
1180	3548.7	1.2
1181	3551.5	1.3
1182	3551.5	1.2

Defected SWCNT Model

Mode Label	FREQ(cm^-1)	Ld (%)
1	29.1	10.0
2	31.5	69.8
3	33.1	76.5
4	39.3	38.3

5	52.7	57.7
6	52.8	53.0
7	54.8	47.5
8	55.4	84.0
9	58.8	74.3
10	59.7	55.4
11	60.2	67.8
12	61.8	65.4
13	65.8	73.9
14	71.0	49.2
15	72.2	82.8
16	73.1	52.8
17	74.7	48.4
18	76.4	83.1
19	80.1	70.3
20	84.6	68.7
21	86.7	70.2
22	90.4	64.8
23	91.7	67.8
24	94.9	41.5
25	102.6	50.2
26	108.0	60.3
27	110.0	72.7
28	112.9	76.9
29	116.4	74.5
30	120.3	76.9
31	126.3	71.4
32	135.7	79.9
33	140.6	75.0
34	152.0	79.6
35	154.6	81.7
36	155.8	69.6
37	157.2	83.3
38	160.2	83.4
39	161.4	86.6
40	162.0	82.1
41	163.9	82.2
42	164.8	81.4
43	166.2	87.8
44	169.8	86.7
45	172.1	86.1
46	176.4	86.1
47	179.5	81.5
48	181.3	83.5
49	183.8	79.5
50	185.7	85.4

51	188.4	83.0
52	190.1	82.9
53	194.7	78.8
54	198.3	83.3
55	199.7	90.1
56	203.1	79.2
57	204.8	84.7
58	205.4	82.9
59	206.2	82.0
60	212.2	77.9
61	214.3	72.8
62	216.0	74.8
63	219.2	75.4
64	222.0	80.3
65	222.9	76.5
66	227.0	70.7
67	229.1	81.4
68	230.4	69.4
69	233.3	65.8
70	235.3	55.9
71	237.8	64.3
72	239.8	44.8
73	240.2	37.7
74	242.0	80.7
75	244.7	55.2
76	246.9	70.3
77	247.8	62.4
78	250.0	74.1
79	252.2	76.8
80	252.8	74.5
81	254.0	83.8
82	254.7	81.0
83	257.9	63.1
84	259.7	74.9
85	263.0	62.0
86	264.2	61.0
87	266.5	69.9
88	267.9	84.8
89	269.2	64.4
90	273.6	84.7
91	276.3	84.6
92	277.3	72.7
93	278.3	86.3
94	279.3	83.7
95	281.7	77.3
96	283.0	64.7

97	284.3	74.4
98	286.1	74.9
99	287.7	77.4
100	288.2	84.1
101	292.3	83.6
102	293.0	73.8
103	294.1	81.5
104	294.8	86.3
105	295.5	90.3
106	298.0	85.9
107	298.8	88.3
108	301.3	88.9
109	302.9	85.5
110	305.7	86.5
111	307.0	86.2
112	307.6	84.8
113	308.1	83.8
114	309.7	86.3
115	310.2	86.6
116	311.9	81.9
117	313.1	79.3
118	314.9	84.9
119	317.3	80.7
120	317.9	87.5
121	322.7	85.1
122	323.0	86.3
123	325.1	84.4
124	325.2	82.0
125	331.6	86.1
126	332.8	86.8
127	333.2	85.6
128	333.9	88.9
129	335.7	86.2
130	336.6	87.8
131	339.4	83.3
132	340.3	88.7
133	341.0	76.8
134	343.1	80.2
135	343.7	71.2
136	346.0	82.0
137	349.5	89.4
138	352.0	86.8
139	352.5	86.8
140	354.3	84.7
141	356.0	85.9
142	356.8	84.1

143	359.5	81.8
144	361.1	83.0
145	364.0	85.1
146	365.1	88.0
147	367.9	87.1
148	369.7	86.9
149	370.2	81.7
150	372.0	82.1
151	375.4	84.0
152	378.6	76.9
153	379.2	84.4
154	380.8	85.6
155	381.2	86.0
156	384.3	86.2
157	386.0	87.3
158	388.1	87.8
159	388.8	89.4
160	390.2	85.9
161	390.5	90.2
162	392.0	89.7
163	393.2	89.3
164	394.4	86.6
165	396.3	90.6
166	397.0	90.3
167	397.9	87.6
168	399.8	89.3
169	401.0	89.0
170	402.0	90.1
171	404.0	89.4
172	405.6	89.7
173	406.2	82.8
174	406.9	91.7
175	408.4	86.4
176	410.0	90.2
177	412.5	87.3
178	413.4	89.9
179	413.9	90.0
180	415.8	90.2
181	416.4	90.8
182	417.7	90.7
183	419.5	90.5
184	421.1	88.6
185	421.8	87.3
186	422.9	87.8
187	424.3	90.2
188	425.2	89.3

189	426.4	83.0
190	427.8	40.1
191	428.3	62.8
192	428.7	79.1
193	429.9	75.9
194	430.2	54.8
195	430.7	65.3
196	431.2	88.4
197	432.4	85.6
198	434.1	75.8
199	434.3	88.0
200	435.7	87.5
201	436.1	89.7
202	436.9	85.9
203	438.3	89.5
204	439.7	89.8
205	440.6	86.9
206	442.4	89.2
207	442.9	90.5
208	445.2	80.6
209	448.2	85.9
210	449.7	87.5
211	450.7	88.5
212	452.5	87.4
213	453.7	88.5
214	453.9	87.2
215	455.3	88.2
216	456.1	86.1
217	457.5	85.2
218	458.0	89.6
219	458.9	91.5
220	459.7	90.1
221	460.6	90.4
222	461.2	89.8
223	462.6	89.1
224	464.3	90.4
225	464.8	91.9
226	465.5	88.0
227	466.4	89.5
228	467.3	88.1
229	468.1	88.3
230	468.5	87.6
231	468.7	85.8
232	469.5	86.4
233	471.6	87.8
234	474.3	86.5

235	475.5	82.7
236	476.8	79.8
237	477.3	88.6
238	478.7	86.4
239	479.1	87.4
240	479.7	76.9
241	481.8	87.2
242	483.7	82.6
243	484.9	86.4
244	487.3	84.7
245	490.4	81.3
246	490.6	79.4
247	492.4	87.2
248	493.2	85.0
249	493.9	80.7
250	497.3	89.7
251	499.2	90.9
252	499.7	91.5
253	500.3	88.5
254	504.1	89.7
255	505.7	86.7
256	508.5	90.7
257	511.0	85.7
258	512.4	89.2
259	513.0	77.8
260	515.5	88.7
261	517.4	85.2
262	518.1	81.8
263	522.0	89.1
264	522.6	88.0
265	525.9	88.4
266	526.8	89.7
267	530.6	89.4
268	531.9	60.8
269	532.9	82.7
270	534.6	84.0
271	535.7	85.7
272	538.6	87.2
273	541.4	84.2
274	542.1	81.6
275	543.8	81.8
276	546.5	92.6
277	547.0	86.9
278	548.2	89.3
279	548.9	83.7
280	550.4	83.4

281	552.5	88.2
282	553.8	89.2
283	558.8	89.7
284	559.9	87.2
285	560.4	89.1
286	563.1	79.2
287	564.9	80.5
288	566.2	66.9
289	567.0	78.8
290	569.3	87.7
291	570.3	89.3
292	571.9	83.9
293	572.9	79.1
294	575.3	89.2
295	576.8	82.7
296	578.2	88.9
297	579.1	84.6
298	580.8	90.5
299	581.6	88.8
300	582.2	88.0
301	583.4	88.4
302	585.3	89.5
303	586.0	91.1
304	587.9	85.2
305	588.5	91.7
306	588.5	81.9
307	589.9	92.4
308	590.3	90.6
309	591.7	89.0
310	592.6	92.0
311	594.0	88.0
312	594.5	91.3
313	595.4	89.8
314	597.0	91.9
315	598.4	88.2
316	601.0	89.8
317	601.5	88.0
318	603.1	87.8
319	604.0	90.1
320	605.7	87.1
321	605.8	85.6
322	606.3	86.7
323	608.2	83.9
324	608.9	83.9
325	609.4	88.1
326	610.9	84.5

327	612.3	90.4
328	612.9	86.9
329	613.4	89.4
330	613.8	86.8
331	614.2	91.9
332	615.4	89.6
333	616.4	88.0
334	616.5	90.2
335	618.5	91.6
336	620.2	93.0
337	620.6	91.3
338	621.5	89.6
339	621.8	91.5
340	622.7	88.2
341	624.1	86.0
342	624.4	83.6
343	625.5	89.4
344	626.8	91.1
345	628.2	90.2
346	629.2	88.7
347	629.4	85.2
348	631.4	90.3
349	631.9	91.1
350	632.8	89.8
351	633.4	91.3
352	634.3	91.6
353	635.2	90.7
354	635.6	88.9
355	636.2	88.9
356	637.2	91.6
357	637.8	92.7
358	639.0	89.1
359	639.4	84.4
360	639.9	91.4
361	640.6	88.0
362	641.4	89.0
363	642.7	89.5
364	643.4	90.4
365	644.4	89.7
366	644.4	89.2
367	645.6	90.4
368	646.1	89.5
369	647.5	86.7
370	648.6	88.6
371	649.2	91.3
372	650.0	89.5

373	650.6	91.2
374	651.9	88.9
375	652.2	89.3
376	653.5	60.0
377	653.7	75.8
378	653.7	71.5
379	655.2	83.4
380	656.0	79.0
381	657.1	75.2
382	657.3	87.5
383	657.4	85.2
384	658.4	84.7
385	659.1	90.9
386	660.0	86.8
387	661.0	86.3
388	662.4	89.7
389	662.9	92.8
390	664.0	87.2
391	664.9	90.9
392	665.8	88.9
393	666.8	83.6
394	666.9	87.0
395	667.3	89.0
396	667.6	87.9
397	668.3	89.8
398	669.6	91.1
399	669.9	87.9
400	671.2	89.3
401	672.2	90.4
402	672.3	85.8
403	672.8	87.6
404	674.8	90.2
405	675.8	89.9
406	676.5	88.2
407	678.4	84.0
408	679.6	89.0
409	681.1	85.0
410	681.9	89.1
411	682.6	89.6
412	684.7	88.1
413	684.9	86.6
414	685.7	82.8
415	687.0	89.3
416	688.4	89.0
417	689.2	90.2
418	690.3	86.3

419	691.0	87.1
420	691.4	90.0
421	691.9	92.9
422	692.8	87.6
423	693.5	88.0
424	696.0	90.0
425	696.5	91.4
426	696.7	87.7
427	697.3	88.1
428	699.5	85.2
429	700.0	89.2
430	700.1	91.2
431	701.2	85.9
432	702.0	88.2
433	703.8	87.6
434	705.1	87.0
435	706.2	87.1
436	707.8	90.0
437	708.2	90.0
438	708.5	85.3
439	709.1	90.4
440	709.4	85.9
441	710.8	89.6
442	712.2	89.4
443	712.7	86.0
444	714.9	88.6
445	715.6	90.0
446	717.0	88.4
447	717.9	88.0
448	718.1	89.2
449	719.4	87.7
450	720.2	82.4
451	721.2	89.4
452	722.5	85.9
453	723.3	87.3
454	724.6	88.9
455	725.9	81.4
456	727.6	90.3
457	727.7	90.3
458	728.7	86.5
459	729.7	86.9
460	730.8	87.4
461	731.9	88.3
462	732.4	85.1
463	734.0	88.4
464	734.6	86.7

465	735.1	81.1
466	736.3	83.8
467	738.1	77.9
468	738.8	87.3
469	739.1	68.6
470	739.7	80.5
471	740.5	70.8
472	742.5	52.1
473	743.1	31.5
474	744.0	71.1
475	744.1	53.9
476	746.0	75.3
477	746.3	80.6
478	746.6	64.8
479	747.8	71.7
480	748.1	69.4
481	749.1	80.2
482	750.1	89.5
483	752.5	80.7
484	752.8	83.5
485	753.0	77.8
486	753.8	84.5
487	756.2	73.6
488	756.8	82.4
489	757.9	87.1
490	758.2	86.1
491	759.9	85.1
492	760.8	77.1
493	761.4	84.6
494	763.9	82.9
495	765.0	77.9
496	766.4	73.5
497	767.7	75.8
498	769.5	75.7
499	771.2	80.4
500	771.7	83.3
501	773.0	74.4
502	773.1	87.5
503	773.9	82.8
504	775.5	76.6
505	776.5	82.0
506	777.7	74.9
507	778.4	82.1
508	778.9	88.3
509	780.3	68.6
510	781.0	78.0

511	781.4	66.6
512	782.8	72.1
513	783.8	84.8
514	785.6	77.7
515	786.3	70.1
516	787.6	89.7
517	787.8	81.4
518	789.2	76.3
519	789.5	83.2
520	790.8	68.4
521	792.2	82.1
522	793.1	68.2
523	793.5	79.2
524	794.5	53.9
525	795.0	65.1
526	795.3	61.7
527	796.3	76.2
528	797.6	77.4
529	798.0	75.4
530	798.2	72.6
531	799.4	77.8
532	799.7	68.2
533	799.9	57.6
534	801.3	81.2
535	801.7	83.8
536	802.2	76.8
537	803.1	77.1
538	804.3	70.7
539	805.1	74.3
540	805.4	61.5
541	806.2	78.4
542	807.3	78.8
543	807.5	81.1
544	808.3	82.6
545	808.5	79.2
546	808.8	84.5
547	810.1	68.7
548	810.7	79.1
549	811.7	77.1
550	812.6	82.2
551	813.5	74.4
552	814.6	71.5
553	815.1	85.3
554	817.6	83.1
555	818.7	87.0
556	821.5	81.8

557	823.0	83.0
558	823.2	88.1
559	824.1	88.6
560	824.9	89.7
561	825.3	89.0
562	825.5	87.4
563	826.2	88.6
564	827.9	81.0
565	828.8	88.5
566	831.1	83.9
567	831.5	79.0
568	832.4	88.6
569	833.5	87.4
570	834.4	83.9
571	834.7	88.2
572	835.4	86.3
573	836.9	88.2
574	837.4	84.9
575	838.0	87.3
576	838.7	85.8
577	839.2	84.1
578	840.1	88.4
579	840.5	91.5
580	841.1	87.8
581	842.2	85.9
582	842.8	83.3
583	844.3	84.0
584	844.6	89.2
585	845.2	90.1
586	845.6	82.7
587	846.6	80.4
588	846.8	78.4
589	847.2	83.6
590	848.5	85.3
591	850.4	82.7
592	851.2	82.5
593	851.6	89.1
594	852.5	85.8
595	853.1	88.2
596	854.7	85.3
597	855.3	84.4
598	855.6	88.9
599	857.2	88.7
600	858.0	83.1
601	858.7	80.2
602	859.3	82.0

603	860.1	85.0
604	860.6	84.7
605	861.0	79.1
606	862.7	74.9
607	862.9	79.5
608	863.0	85.6
609	863.4	91.2
610	863.7	88.0
611	863.8	80.2
612	865.2	90.1
613	866.0	72.3
614	866.0	68.0
615	866.8	76.4
616	867.3	68.5
617	867.4	88.9
618	869.7	88.1
619	871.6	90.3
620	872.8	87.7
621	877.0	87.9
622	878.8	86.3
623	883.2	87.9
624	888.2	83.4
625	889.9	87.3
626	892.3	85.1
627	895.2	72.1
628	896.8	75.3
629	897.3	83.3
630	899.9	22.7
631	900.1	60.9
632	901.3	41.3
633	904.7	36.9
634	905.2	35.6
635	905.7	74.2
636	906.0	9.2
637	910.2	87.5
638	914.5	77.9
639	918.9	23.9
640	919.4	18.4
641	922.1	78.6
642	923.1	66.4
643	926.8	78.7
644	927.4	68.4
645	930.0	32.0
646	930.4	11.8
647	932.4	77.1
648	933.4	77.7

649	937.5	16.1
650	938.0	21.5
651	938.6	52.9
652	939.7	55.3
653	943.7	79.0
654	946.5	53.1
655	948.7	32.6
656	950.4	29.0
657	950.7	83.6
658	952.7	45.9
659	954.4	31.7
660	955.0	13.4
661	956.1	36.6
662	958.7	43.1
663	960.1	8.1
664	960.4	8.2
665	960.9	19.2
666	961.5	38.4
667	962.6	10.8
668	962.7	11.0
669	965.1	59.4
670	966.2	16.4
671	967.1	22.4
672	967.8	72.4
673	969.9	75.0
674	971.0	62.7
675	971.8	58.2
676	975.4	82.5
677	978.6	42.3
678	978.9	76.1
679	980.9	39.2
680	982.3	47.8
681	987.3	75.4
682	991.8	74.8
683	993.7	74.3
684	994.8	81.6
685	997.1	83.4
686	998.1	89.7
687	1003.8	82.9
688	1006.3	87.0
689	1008.4	87.7
690	1015.2	74.7
691	1017.9	49.6
692	1019.6	44.9
693	1023.2	76.2
694	1025.4	74.8

695	1026.3	65.1
696	1031.4	62.4
697	1034.1	49.7
698	1038.0	78.0
699	1039.3	66.0
700	1042.1	15.3
701	1042.3	59.2
702	1043.2	9.3
703	1048.4	81.8
704	1050.8	71.8
705	1051.0	75.1
706	1053.2	85.0
707	1053.3	79.8
708	1056.5	10.5
709	1056.9	15.0
710	1057.4	10.9
711	1057.7	22.1
712	1058.8	82.5
713	1064.5	64.3
714	1067.8	80.3
715	1072.8	76.8
716	1073.7	84.3
717	1077.8	88.0
718	1084.0	76.0
719	1087.4	80.5
720	1093.1	87.0
721	1094.4	88.4
722	1099.3	82.0
723	1103.1	84.6
724	1107.5	15.5
725	1107.8	29.6
726	1110.5	80.2
727	1111.3	72.3
728	1112.9	79.2
729	1115.7	81.0
730	1116.9	83.3
731	1119.7	80.5
732	1126.2	87.1
733	1134.4	88.8
734	1137.2	77.0
735	1140.8	78.7
736	1143.2	87.7
737	1145.5	76.4
738	1149.6	81.6
739	1151.3	88.1
740	1154.5	75.4

741	1155.8	77.4
742	1158.8	72.9
743	1163.6	39.6
744	1164.3	80.2
745	1166.3	56.4
746	1167.7	53.3
747	1169.1	62.7
748	1172.2	60.2
749	1174.7	65.8
750	1176.8	69.7
751	1178.7	75.6
752	1181.5	80.1
753	1184.1	68.1
754	1188.4	78.0
755	1190.5	75.5
756	1192.6	69.1
757	1193.7	83.0
758	1196.7	81.3
759	1198.9	76.0
760	1200.8	74.9
761	1206.2	73.1
762	1207.4	77.0
763	1210.5	76.0
764	1212.2	69.7
765	1216.2	72.7
766	1217.9	42.6
767	1219.3	67.9
768	1220.4	66.6
769	1222.9	77.2
770	1223.0	79.2
771	1224.8	73.1
772	1226.4	68.4
773	1230.6	74.3
774	1231.5	73.8
775	1233.8	72.6
776	1234.9	55.4
777	1237.7	79.5
778	1238.7	59.5
779	1241.0	64.4
780	1243.4	26.3
781	1243.6	12.3
782	1244.2	57.1
783	1244.7	57.4
784	1248.4	61.1
785	1249.4	75.2
786	1251.8	81.6

787	1253.4	83.5
788	1255.1	78.0
789	1258.5	74.9
790	1260.3	79.0
791	1262.2	74.3
792	1263.0	83.1
793	1264.0	71.9
794	1265.5	78.0
795	1266.2	75.7
796	1268.9	73.0
797	1269.7	14.0
798	1270.0	69.5
799	1270.5	68.3
800	1270.9	11.3
801	1274.5	81.5
802	1276.5	81.7
803	1278.8	82.8
804	1279.3	67.8
805	1281.3	77.2
806	1282.2	84.6
807	1282.3	68.6
808	1284.2	71.2
809	1286.7	71.8
810	1287.6	77.6
811	1287.9	82.4
812	1291.3	80.3
813	1293.5	80.4
814	1297.1	77.1
815	1298.2	69.9
816	1299.8	60.9
817	1302.2	68.9
818	1304.9	78.6
819	1306.3	75.3
820	1308.0	68.0
821	1311.2	77.7
822	1311.9	80.9
823	1314.8	81.0
824	1315.6	69.1
825	1316.9	76.3
826	1317.6	76.1
827	1320.6	78.6
828	1321.9	79.7
829	1323.0	85.5
830	1325.2	82.0
831	1325.8	73.8
832	1326.0	80.8

833	1327.6	75.3
834	1328.9	46.5
835	1330.1	66.8
836	1331.4	54.6
837	1332.5	76.8
838	1333.2	78.3
839	1334.0	63.4
840	1335.8	71.0
841	1336.7	59.4
842	1338.7	76.4
843	1340.3	76.6
844	1341.3	81.1
845	1341.5	81.2
846	1342.8	87.8
847	1343.0	85.6
848	1344.9	80.0
849	1346.1	80.2
850	1347.7	68.3
851	1349.2	85.7
852	1352.9	84.0
853	1353.6	87.5
854	1354.1	89.8
855	1356.5	89.5
856	1357.4	83.5
857	1359.0	89.3
858	1361.7	83.5
859	1362.6	82.8
860	1363.0	84.5
861	1364.7	88.4
862	1366.7	89.4
863	1367.2	86.8
864	1368.5	82.5
865	1369.8	85.5
866	1371.6	84.9
867	1373.0	89.4
868	1374.4	82.4
869	1374.9	69.7
870	1376.4	82.4
871	1378.1	84.4
872	1380.5	84.0
873	1381.1	76.8
874	1381.8	82.4
875	1384.0	87.5
876	1386.7	90.5
877	1388.1	85.3
878	1388.3	85.4

879	1390.6	85.6
880	1391.4	89.7
881	1393.2	88.3
882	1393.5	82.3
883	1395.0	84.8
884	1396.1	87.3
885	1397.2	91.1
886	1399.0	87.7
887	1399.2	81.9
888	1400.6	83.1
889	1401.2	89.5
890	1402.0	80.6
891	1402.4	85.8
892	1402.9	87.0
893	1404.0	89.3
894	1404.4	87.7
895	1405.7	87.6
896	1406.2	85.2
897	1407.2	89.1
898	1408.5	81.8
899	1409.5	86.4
900	1410.8	88.2
901	1411.6	86.4
902	1412.7	61.0
903	1413.7	86.5
904	1414.0	82.3
905	1414.7	85.5
906	1415.6	58.7
907	1416.6	68.1
908	1417.2	88.1
909	1417.7	72.1
910	1418.3	25.4
911	1418.9	78.3
912	1419.6	54.8
913	1420.0	70.9
914	1421.2	78.1
915	1421.8	83.6
916	1422.4	79.8
917	1422.7	88.0
918	1422.7	87.1
919	1423.6	84.0
920	1424.4	85.9
921	1424.5	86.4
922	1425.3	82.8
923	1425.7	87.1
924	1427.0	86.8

925	1427.3	88.6
926	1427.9	87.6
927	1428.2	85.4
928	1429.3	88.0
929	1429.5	86.0
930	1430.2	87.8
931	1431.2	87.6
932	1431.7	88.0
933	1432.7	83.2
934	1433.2	83.0
935	1433.5	84.9
936	1434.1	85.9
937	1434.8	86.4
938	1435.0	87.2
939	1435.8	89.3
940	1436.3	87.6
941	1436.6	83.4
942	1437.2	86.6
943	1437.7	84.7
944	1437.8	87.7
945	1438.6	83.1
946	1439.0	83.4
947	1439.8	86.4
948	1440.5	86.6
949	1440.7	87.8
950	1441.7	84.9
951	1442.3	87.7
952	1442.9	87.0
953	1443.4	86.0
954	1443.6	90.9
955	1444.2	85.7
956	1444.7	85.1
957	1445.1	89.5
958	1445.7	80.4
959	1446.3	86.7
960	1446.6	88.5
961	1446.8	86.2
962	1447.5	89.4
963	1448.8	83.3
964	1449.4	88.7
965	1450.3	88.8
966	1450.7	76.8
967	1451.3	87.7
968	1451.5	90.0
969	1452.3	85.1
970	1452.7	87.5

971	1453.2	87.5
972	1453.5	87.3
973	1454.9	88.1
974	1455.4	85.1
975	1455.6	88.0
976	1456.7	86.7
977	1457.3	89.4
978	1458.6	88.5
979	1459.5	85.4
980	1459.9	86.0
981	1461.5	87.2
982	1461.9	79.4
983	1462.2	84.4
984	1462.8	84.4
985	1464.3	85.4
986	1465.2	83.3
987	1465.6	86.2
988	1466.3	78.5
989	1467.3	79.2
990	1468.5	80.9
991	1469.1	87.9
992	1471.0	91.6
993	1471.3	86.3
994	1471.7	87.4
995	1473.3	86.6
996	1474.0	90.1
997	1474.4	89.3
998	1475.1	82.9
999	1476.2	85.6
1000	1477.8	86.5
1001	1478.3	86.4
1002	1479.8	89.7
1003	1480.6	90.5
1004	1481.6	85.2
1005	1481.8	80.5
1006	1483.2	89.0
1007	1483.9	87.6
1008	1484.3	87.3
1009	1485.4	89.3
1010	1485.7	79.8
1011	1487.4	90.6
1012	1489.3	89.4
1013	1489.8	89.3
1014	1490.7	92.2
1015	1491.9	87.6
1016	1493.3	83.9

1017	1494.8	89.5
1018	1495.5	79.1
1019	1497.8	89.2
1020	1498.1	88.8
1021	1499.3	85.9
1022	1500.0	88.4
1023	1500.7	89.6
1024	1502.5	91.0
1025	1503.1	90.0
1026	1503.9	85.0
1027	1505.4	90.6
1028	1506.7	84.8
1029	1507.6	91.0
1030	1508.5	90.0
1031	1510.7	85.5
1032	1511.7	85.7
1033	1512.3	80.7
1034	1513.3	84.7
1035	1514.3	90.7
1036	1515.5	88.9
1037	1517.4	85.8
1038	1518.6	90.1
1039	1519.4	85.7
1040	1520.1	86.8
1041	1521.6	88.1
1042	1522.8	90.2
1043	1523.2	85.5
1044	1524.1	90.5
1045	1526.4	87.0
1046	1527.4	84.9
1047	1528.1	86.4
1048	1528.9	73.6
1049	1530.5	85.7
1050	1532.1	89.4
1051	1532.2	83.0
1052	1532.9	84.0
1053	1535.1	91.2
1054	1535.9	74.6
1055	1537.3	86.7
1056	1537.5	83.1
1057	1539.2	82.2
1058	1539.6	78.3
1059	1540.4	76.5
1060	1541.1	67.3
1061	1541.6	81.1
1062	1542.5	80.0

1063	1543.1	83.2
1064	1543.7	79.8
1065	1544.4	82.2
1066	1546.3	80.9
1067	1547.3	71.4
1068	1548.5	84.3
1069	1548.9	66.1
1070	1549.1	66.7
1071	1549.8	85.1
1072	1550.4	55.9
1073	1551.1	41.0
1074	1552.7	55.7
1075	1552.9	67.9
1076	1553.6	68.3
1077	1554.2	65.6
1078	1554.4	62.8
1079	1555.3	52.8
1080	1555.9	72.5
1081	1556.7	52.8
1082	1557.7	67.7
1083	1558.0	69.4
1084	1559.5	56.8
1085	1560.1	59.4
1086	1561.7	73.1
1087	1562.7	63.3
1088	1563.7	58.4
1089	1564.7	70.5
1090	1565.5	24.4
1091	1565.7	26.6
1092	1568.5	84.9
1093	1569.0	69.8
1094	1569.6	61.1
1095	1570.5	86.1
1096	1572.3	75.9
1097	1574.1	87.5
1098	1575.0	84.8
1099	1576.2	79.5
1100	1579.6	79.1
1101	1580.8	84.7
1102	1581.5	80.5
1103	1583.0	91.3
1104	1585.8	89.0
1105	1586.8	90.2
1106	1587.7	88.6
1107	1589.9	81.5
1108	1590.4	84.3

1109	1591.4	85.6
1110	1593.4	80.3
1111	1596.4	86.9
1112	1597.7	86.7
1113	1599.6	89.8
1114	1599.8	89.6
1115	1602.1	89.1
1116	1603.8	86.1
1117	1605.1	86.6
1118	1606.7	79.5
1119	1608.5	89.7
1120	1609.3	90.0
1121	1609.8	89.8
1122	1610.3	85.3
1123	1613.5	87.2
1124	1614.2	85.7
1125	1614.6	81.0
1126	1615.9	84.8
1127	1616.3	79.4
1128	1618.1	53.8
1129	1619.5	12.1
1130	1619.7	33.5
1131	1621.3	86.5
1132	1622.6	85.6
1133	1624.6	88.3
1134	1625.2	89.7
1135	1626.7	86.4
1136	1627.0	67.2
1137	1628.8	85.1
1138	1629.8	74.4
1139	1631.2	83.6
1140	1631.8	83.1
1141	1632.1	86.3
1142	1634.8	86.1
1143	1635.2	74.3
1144	1637.7	84.8
1145	1640.6	89.4
1146	1642.0	85.7
1147	1646.5	89.9
1148	1649.6	86.8
1149	1651.1	42.0
1150	1651.2	42.9
1151	1652.4	86.9
1152	1653.7	82.6
1153	1655.8	83.6
1154	1656.9	87.0

1155	1657.0	87.5
1156	1658.6	86.0
1157	1659.5	86.5
1158	1660.6	83.4
1159	1660.7	83.0
1160	1661.6	83.4
1161	1662.0	79.4
1162	1662.5	85.2
1163	1663.1	87.8
1164	1663.5	89.3
1165	1664.2	86.9
1166	1664.6	87.1
1167	1665.4	75.7
1168	1666.3	86.8
1169	1666.6	84.1
1170	1667.2	86.2
1171	1668.3	79.3
1172	1669.0	89.4
1173	1669.9	64.0
1174	1670.0	79.1
1175	1671.4	85.4
1176	1671.5	73.3
1177	1673.4	86.1
1178	1675.0	84.6
1179	1675.9	83.1
1180	1677.1	84.7
1181	1677.6	83.7
1182	1678.1	86.8
1183	1680.3	83.1
1184	1681.4	86.7
1185	1682.3	87.2
1186	1683.4	81.2
1187	1684.1	83.3
1188	1686.0	86.0
1189	1687.0	81.1
1190	1687.6	79.1
1191	1689.0	85.4
1192	1689.5	62.3
1193	1690.2	63.9
1194	1691.0	76.8
1195	1692.5	67.9
1196	1692.8	55.0
1197	1694.2	74.7
1198	1695.4	74.8
1199	1697.9	21.6
1200	1698.0	20.3

1201	1709.7	21.3
1202	1709.7	21.4
1203	1719.9	8.2
1204	1720.0	8.4
1205	1737.9	9.2
1206	1738.9	9.1
1207	1758.2	6.1
1208	1758.3	4.4
1209	1800.2	4.1
1210	1800.2	3.7
1211	1811.1	3.5
1212	1811.2	3.5
1213	3394.3	1.8
1214	3394.5	3.3
1215	3394.8	2.0
1216	3394.8	2.1
1217	3395.1	2.2
1218	3395.2	4.2
1219	3446.3	2.9
1220	3446.4	3.1
1221	3446.4	2.5
1222	3446.5	3.0
1223	3447.9	5.4
1224	3448.0	5.6
1225	3450.6	1.4
1226	3450.7	1.4
1227	3451.5	2.1
1228	3451.5	2.2
1229	3452.1	2.3
1230	3452.2	2.2
1231	3452.4	1.3
1232	3452.4	1.3
1233	3458.5	4.5
1234	3458.6	4.1
1235	3459.4	1.4
1236	3459.4	1.4
1237	3469.2	6.1
1238	3469.3	6.4
1239	3477.4	5.9
1240	3477.6	6.0
1241	3481.6	3.5
1242	3481.6	4.8
1243	3548.1	1.2
1244	3548.2	1.2
1245	3548.7	1.2
1246	3548.8	2.0

1247	3551.3	1.2
1248	3551.5	1.3
